# Fungi in Asthma: From Epidemiology and Airway Mycobiome to Molecular Mechanisms and Novel Allergenic Phytopathogens

**DOI:** 10.3390/ijms27198639

**Published:** 2026-09-27

**Authors:** Magdalena Krasowska-Kunach, Michał Sułek, Agnieszka Szuster-Ciesielska

**Affiliations:** Department of Virology and Immunology, Institute of Biological Sciences, Faculty of Biology and Biotechnology, Maria Curie-Skłodowska University, Akademicka 19 St., 20-033 Lublin, Poland; magda30krasowska@gmail.com (M.K.-K.); michal.sulek@umcs.pl (M.S.)

**Keywords:** airway mycobiome, fungal sensitization, type 2 inflammation, severe asthma with fungal sensitization, allergic bronchopulmonary aspergillosis, pattern recognition receptors, epithelial alarmins, phytopathogenic microfungi, gut–lung axis, precision medicine

## Abstract

Fungal exposure and sensitization are linked to asthma severity, but their clinical significance varies by host, exposure context, and disease phenotype. This review integrates epidemiological, immunological, and clinical evidence on fungal-associated asthma, distinguishing environmental exposure, allergic sensitization, and persistent airway colonization. We examine how fungal allergens, proteases, and cell-wall components engage pattern-recognition receptors, disrupt epithelial barriers, and activate overlapping type 2 and type 17 inflammatory pathways. Studies of the airway and intestinal mycobiome offer additional perspectives on host–fungal interactions, although most human findings remain associative and do not establish causality or clinical utility. Severe asthma with fungal sensitization (SAFS) and allergic bronchopulmonary aspergillosis (ABPA) are distinct clinical contexts that require different diagnostic and therapeutic approaches. Antifungal treatment has an established role in ABPA, whereas evidence in SAFS is inconsistent; biologic selection should be guided by clinical phenotype and established eligibility criteria rather than fungal sensitization alone. We also synthesize our published biochemical, epithelial-cell, and murine studies of seven plant-associated fungal and oomycete taxa from eastern Poland. These findings support further investigation of their allergenic and pro-inflammatory potential but do not establish clinical allergenicity or predict treatment response in humans. Priorities include standardized exposure assessment, validated diagnostic reagents, and longitudinal studies linking fungal measurements to clinical outcomes. Expanded allergen panels and mycobiome-based approaches require prospective validation before routine clinical implementation.

## 1. Introduction

Asthma is a heterogeneous inflammatory airway disease in which environmental exposures contribute to differences in clinical course [1]. Fungal-associated disease is particularly relevant in patients with severe symptoms. Allergic bronchopulmonary aspergillosis (ABPA) illustrates how airway exposure to fungi can be associated with a distinct clinical syndrome that requires specific assessment [2]. The broader relationship between fungi and asthma extends beyond ABPA and includes sensitization to airborne molds.

Sensitization to *Alternaria alternata* has been linked to respiratory arrest in young patients with asthma, and high outdoor mold concentrations have been associated with asthma mortality [3,4]. In the European Community Respiratory Health Survey, sensitization to *A. alternata* or *Cladosporium herbarum* was more strongly associated with severe asthma than sensitization to several other common aeroallergens [5]. Studies of indoor mold and dampness also support an association with asthma development and exacerbations [6,7,8]. SAFS (Severe Asthma with Fungal Sensitisation) is defined as severe asthma with sensitization to one or more fungi after exclusion of ABPA; fungal sensitization is associated with greater disease severity [9,10]. Exposure, sensitization, and airway colonization should not be treated as interchangeable findings.

Sequencing studies have identified differences in sputum fungal communities between patients with asthma and non-atopic controls [11]. Fungal and bacterial dysbiosis in infancy has also been linked to subsequent atopic wheeze, suggesting that intestinal microbial communities may influence respiratory outcomes [12]. These observations extend the clinical question beyond inhaled allergens to the potential contribution of resident fungal communities. They do not establish whether altered fungal composition causes airway disease or merely reflects inflammation and treatment effects. Distinguishing these possibilities is important when interpreting mycobiome associations as potential therapeutic targets.

Clinical evidence supports investigating fungal contributions to difficult-to-treat asthma, but sensitization alone does not identify the mechanism sustaining disease. In the FAST trial, itraconazole improved asthma-related quality of life in a subset of patients with SAFS [13]. This finding supports further study of antifungal treatment in selected patients, but does not establish a general indication for fungal-sensitized asthma. A mechanistic explanation is also needed for the variable clinical consequences of fungal exposure. Experimental studies show that *Alternaria* serine protease activity can trigger rapid airway IL-33 release and exacerbate inflammation in mice [14]. These findings provide testable links between fungal products and epithelial immune responses, while requiring careful distinction from evidence obtained in patients.

This review examines the immunological mechanisms linking fungal exposure and sensitization to severe asthma, with an emphasis on their relevance to clinical management. It also considers plant-associated microfungi investigated by our group in the Polish agricultural environment, which broaden the range of fungal exposures examined in asthma research. The aim is to identify where mechanistic evidence can explain clinical observations and where conclusions remain dependent on experimental models.

## 2. Asthma Epidemiology and Genetics Relevant to Fungal Exposure

Asthma affected approximately 260.5 million people worldwide in 2021. Although age-standardized prevalence has declined since 1990, absolute case numbers continue to rise with population growth and aging [15,16,17]. Prevalence varies several-fold across and within regions, in both pediatric surveys and adult cohorts such as the European Community Respiratory Health Survey [18,19,20,21,22,23]. Central and Eastern European surveillance shows the same pattern: Polish programs, including PMSEAD and ECAP; regional studies from Upper Silesia; and the BUPAS cohort all documented a substantial symptom burden alongside marked under-recognition of physician-diagnosed disease [24,25,26,27]. A similar pattern emerged in Austria, where estimated prevalence depended strongly on the diagnostic definition used [28]. Climate, housing quality, occupational setting, and regional aeroallergen profiles shape the intensity and taxonomic composition of fungal exposure, so this heterogeneity directly translates into fungal asthma.

Prevalence is higher in boys during childhood and reverses after puberty. In adults, women report worse symptom control and greater health-care use, and are over-represented among late-onset, eosinophilic, and severe phenotypes [29,30,31,32,33]. Adult-onset disease is more often associated with obesity, smoking, occupational exposure, chronic rhinosinusitis, and less reversible airflow obstruction, and has a partially distinct genetic architecture from childhood-onset asthma [34,35]. In older adults, asthma is frequently misattributed to chronic obstructive pulmonary disease or heart failure, and immunosenescence and multimorbidity alter treatment response [36,37,38]. Fungal sensitization and airway colonization are enriched among patients whose asthma remains uncontrolled despite guideline-based therapy.

Asthma heritability is substantial, but no single variant is determinative [39]. Genome-wide association studies have identified reproducible susceptibility regions, most prominently 17q12–21 in childhood-onset disease, along with loci involving HLA class II, *IL33*, *IL1RL1/IL18R1*, *TSLP*, and the type 2 cytokine cluster containing *IL13.* Trans-ancestry analyses have expanded this catalog and confirmed ancestry-dependent effects [40,41,42,43,44]. Variants in *FLG* and other epithelial-barrier genes most closely track atopic trajectories [42,45,46,47]. Polygenic scores remain insufficiently predictive for individual clinical use. Genetic effects are conditional on exposure: associations at 17q21 are modified by early-life respiratory infection and by microbial or farming environments [48,49], and damp housing, visible mold, occupational bioaerosols, pollution, and tobacco smoke influence epithelial integrity and immune maturation [49,50].

Associations between HLA class II variation and ABPA have been reported, but these findings do not establish a clinically useful genetic predictor. The contribution of host genetics to fungal-associated asthma remains insufficiently defined and requires further investigation in well-characterized patient cohorts [51,52,53].

## 3. Immunological and Molecular Mechanisms of Fungal-Driven Asthma

Fungal exposure of the airway differs from transient aeroallergen challenge: germinating spores and persistent colonization deliver allergens alongside pattern-recognition ligands over prolonged periods [54,55,56]. These mechanisms operate across a disease spectrum extending from SAFS to allergic bronchopulmonary mycosis and chronic airway colonization (Figure 1).

Airway epithelial cells, alveolar macrophages, and dendritic cells detect spores and hyphal fragments via complementary pattern-recognition receptors (PRRs). C-type lectin receptors, primarily Dectin-1, Dectin-2, and Mincle, recognize fungal glycans, while TLR2 and TLR4 provide signals that modulate cytokine output and adaptive polarization [57,58,59,60,61]. Dectin-1 is the best-characterized of these and binds β-glucans that become progressively exposed as *Aspergillus fumigatus* conidia swell and germinate [62,63]. HemITAM ligation activates Syk and the CARD9–Bcl10–MALT1 complex, driving NF-κB-dependent production of TNF, IL-6, IL-1β, and IL-23 [64,65], a cytokine profile that supports Th17 polarization and, under sustained exposure, mixed Th2/Th17 inflammation with reduced corticosteroid responsiveness. Airway administration of β-glucan enhances hyperresponsiveness via Dectin-1–Syk signaling [65,66,67]. Dectin-2 and Mincle recognize high-mannose glycans and fungal glycolipids, signal through Fcγ-associated ITAM motifs, and shape IL-17 responses in experimental *Malassezia* and *Aspergillus* infection [68,69,70,71,72,73]. Cooperative signaling between lectin receptors and TLR2/TLR4 integrates Syk–CARD9 with MyD88-dependent pathways and alters the balance among clearance, regulation, and inflammation [74,75,76,77,78]. Epithelial cells contribute soluble recognition through antimicrobial peptides, pentraxin 3, collectins, and complement components [79,80,81], and couple recognition to adaptive immunity by releasing IL-33, TSLP, and IL-25 [82,83]. Human genetic evidence is largely confined to severe CARD9 deficiency, which predisposes to invasive and chronic mucocutaneous infection, not to asthma [72,84]. Whether subtler variation in these pathways modifies susceptibility to SAFS or allergic bronchopulmonary mycosis (ABPM) remains untested [53,85,86].

Dendritic cells carrying fungal antigen to draining lymph nodes drive Th2 and Th17 differentiation in parallel [56,87]. Against a background of epithelial alarmins, IL-4 promotes GATA3- and STAT6-dependent Th2 commitment and, together with CD40–CD40L signaling, licenses class switching to IgE; IL-13-producing Tfh13 cells support high-affinity IgE in germinal centers. Th2 cells and ILC2s then sustain IgE synthesis, eosinophil survival, goblet-cell metaplasia, and hyperresponsiveness through IL-4, IL-5, and IL-13 [54,88,89,90,91]. Challenge with *Alternaria*, *Cladosporium*, or *Aspergillus* allergens rapidly induces these cytokines, and their neutralization attenuates eosinophilic inflammation [88,90]. PRR-derived IL-1β, IL-6, and IL-23 promote Th17 differentiation and IL-17A/IL-17F production, recruit neutrophils, and induce epithelial and stromal chemokines [92,93,94,95]. Hybrid Th2/Th17 cells producing both cytokine sets have been identified in SAFS [92,96], and their relative contribution shifts during exacerbation, so individual patients may show concurrent eosinophilic and neutrophilic inflammation. Whether IL-17 signaling causes corticosteroid resistance in human fungal asthma remains unresolved.

Fungus-specific IgE bound to FcεRI on mast cells and basophils enables rapid activation upon re-exposure, triggering degranulation that releases histamine, leukotrienes, prostaglandins, and chemokines, which cause bronchoconstriction, vascular leakage, and further granulocyte recruitment [89,97,98]. Long-lived Th2/Th17 cells, plasma cells, and memory B cells lower the recall threshold, so intermittent low-dose exposure can sustain exacerbations, although no longitudinal human study has yet linked these signatures to clinical trajectory [99,100,101,102].

In fungal-driven asthma, inflammation is coupled to epithelial barrier failure and progressive remodeling. Spores, hyphae, proteases, and oxidative products induce TSLP, IL-33, and IL-25, which are elevated in bronchial biopsies, sputum, and lavage fluid from patients with asthma and correlate with severity and exacerbation frequency [103,104,105,106,107,108]. These alarmins activate dendritic cells, ILC2s, Th2 cells, eosinophils, mast cells, and basophils, and indirectly reinforce Th17-associated inflammation through the surrounding cytokine network [106,107,109]. They also disrupt tight-junction integrity and promote epithelial-to-mesenchymal transition, generating fibroblast-like cells that deposit extracellular matrix. TSLP and IL-33 increase collagen and fibronectin expression in vitro, while IL-25 drives fibroblast proliferation and myofibroblast differentiation, linking recurrent injury to thickening of the subepithelial lamina reticularis [82,110,111].

IL-4 and IL-13 from Th2 cells and ILC2s induce MUC5AC expression and goblet-cell differentiation, producing viscous plugs that contain extracellular DNA, eosinophil granule proteins, and cellular debris [112,113,114,115]. Retained fungal material within these plugs perpetuates epithelial activation, establishing a local positive-feedback loop [116]. TSLP and IL-33 also promote smooth-muscle proliferation and contractility, and bidirectional signaling between smooth muscle and mast cells enhances mast-cell survival while increasing smooth-muscle responsiveness [82,112,117,118]. This increased wall thickness contributes to fixed airflow limitation and reduced bronchodilator reversibility [119]. Alarmin-dependent fibroblast and endothelial activation add collagen deposition and VEGF-associated vascular remodeling, alongside TGF-β released by eosinophils and mast cells [112,120,121,122,123].

Fungal proteases, mycotoxins, cell-wall polysaccharides, lipid mediators, and protein allergens translate exposure into injury and immune activation. They occur in mixtures whose composition varies by species, developmental stage, substrate, temperature, and humidity, so a single exposure may combine barrier disruption, innate receptor activation, metabolic toxicity, and allergen-specific recognition. Protease-rich extracts of *A. fumigatus* and *Alternaria alternata* cause epithelial desquamation, disrupt occludin and claudins, and increase paracellular permeability [124,125,126]; the protease allergen Pen c 13 induces hyperresponsiveness and eosinophilic inflammation in experimental models [127,128]. PAR-2 cleavage triggers calcium signaling and releases IL-6, IL-8, GM-CSF, IL-33, and TSLP [14,129,130], and proteolysis may expose cryptic determinants or generate neo-epitopes [131,132].

Aflatoxins, ochratoxin *A, trichothecenes*, and gliotoxin have been detected in heavily contaminated indoor environments and, in experimental systems, cause oxidative stress, mitochondrial injury, DNA damage, and impaired epithelial repair [133,134,135]; gliotoxin also suppresses NF-κB-dependent activation and phagocyte function, which could favor persistent colonization [133,136]. Exposure assessment of inhaled mycotoxins remains the principal obstacle to interpreting these findings clinically. Chitin fragments induce epithelial CCL2 and CCR2-dependent recruitment of monocytes and eosinophils [137], whereas germination-exposed β-glucans drive Dectin-1-dependent IL-1β and IL-23 production and downstream IL-17 responses [138,139]. Outcomes depend on particle size, solubility, dose, growth stage, and accompanying microbial signals, and most quantitative data derive from purified preparations rather than environmental aerosols [56,140,141].

Fungal prostaglandin-like molecules and oxylipins modulate host inflammatory responses [142,143,144], and fungal exposure alters host arachidonic acid metabolism. Cysteinyl leukotrienes enhance bronchoconstriction, and prostaglandin D2 recruits Th2 cells, ILC2s, and eosinophils through CRTH2 [143,145,146,147]. Classical allergens such as Alt a 1, Asp f proteins, and Pen c 13 combine IgE-binding capacity with enzymatic activity, disrupting barriers, activating complement, or cleaving host proteins, including fibrinogen, to generate danger signals that engage TLR4 [125,126,128,130,131,132,148,149,150,151].

## 4. The Respiratory and Gut Mycobiome in Asthma

The respiratory and intestinal mycobiomes provide complementary perspectives on fungal involvement in asthma. Airway studies examine associations between fungal communities and disease [152,153], whereas gut studies investigate potential systemic immune effects through the gut–lung axis (Figure 2A) [154,155]. Both fields require careful distinction between microbial detection, persistent colonization, and causally demonstrated effects.

### 4.1. Composition of the Airway Mycobiome in Health and Asthma

The healthy airway harbors a low-biomass yet metabolically active fungal community, now regarded as part of the respiratory microbiome rather than contamination from the oropharynx or ambient air [152]. ITS1/ITS2 surveys of nasal, sputum, and bronchoscopic samples identify *Malassezia*, *Candida*, *Cladosporium*, *Aspergillus*, and *Penicillium* as dominant commensal genera, with substantial inter-individual variation and niche-specific differences between the upper and lower airways [156,157]. Evidence on *Malassezia* is conflicting: higher upper-airway *M. globosa* abundance was associated with lower exacerbation risk in one cohort, whereas antibiotic-induced *Malassezia* expansion in the infant gut drives immune dysregulation and airway inflammation in mice, and higher intestinal *Malassezia* is prospectively linked to allergic disease in children [158,159]. The role of these species likely depends on anatomical niche, host age, and coexisting microbiota [160]. In healthy subjects, the community comprises a small set of moderately abundant taxa superimposed on a long tail of transient environmental species, yielding high between-subject beta-diversity [152,161].

Asthmatic upper and lower airways show reproducible compositional shifts, with enrichment of specific filamentous fungi and yeasts and altered diversity [55]. ITS2 sequencing of induced sputum and bronchial brushings has resolved more than 200 species, among which *Aspergillus fumigatus*, *Candida albicans*, and *Mycosphaerella tassiana* are the most prevalent, with relative abundance varying by asthma status and severity. Members of the *Aspergillus niger* complex, such as *Aspergillus tubingensis*, are enriched in protected bronchial brushings and associate with distinct inflammatory profiles, including lower sputum neutrophilia. Thus, a higher *Aspergillus* signal does not map onto a single inflammatory endotype [153]. Species of the *Cryptococcus humicola* complex, including *Cryptococcus pseudolongus*, have been reported as more abundant in bronchoscopy samples from asthmatics, suggesting that shifts in lesser-known basidiomycetous yeasts may also matter [152,153]. *Alternaria*, *Cladosporium* and *Penicillium*, long implicated in mold-related asthma, are detected across both compartments and are more frequent or more abundant in poorly controlled and severe disease. Sequencing studies identify associations between airway fungal profiles and asthma outcomes, but detection of fungal DNA alone does not establish viable or persistent colonization [160,162]. A cross-sectional study found differences in nasal fungal community composition between individuals with allergic rhinitis and/or asthma and healthy controls [161]. Fungal richness tends to be higher in asthma, although the direction of alpha-diversity change differs across studies, reflecting sampling site, sequencing target, bioinformatic pipeline, and environmental exposure [55].

The asthmatic airway mycobiome does not mirror the oropharyngeal or environmental mycobiota. Parallel sampling of sputum, mouthwash, and outdoor air reveals compositionally distinct airway communities enriched for *Aspergillus*, *Candida*, and *Cryptococcus* taxa that are underrepresented in inhaled air and oral samples [153]. Fungal dysbiosis commonly co-occurs with bacterial shifts, including enrichment of Proteobacteria, and upper-airway fungal and bacterial diversity correlate positively, indicating coordinated ecological restructuring [163]. Despite small sample sizes and methodological heterogeneity, the data converge on a model in which overrepresentation of *Aspergillus*, *Candida*, *Alternaria*, and *Cladosporium*, together with depletion or instability of commensal *Malassezia*, characterizes a subset of asthma patients and tracks with impaired control, exacerbation risk, and distinct inflammatory phenotypes.

### 4.2. The Gut Mycobiome, Fungal Metabolites, and the Gut–Lung Axis

The gut mycobiome is a numerically minor yet immunologically potent component of the intestinal ecosystem, dominated by Ascomycota and Basidiomycota and including *Candida*, *Saccharomyces*, *Malassezia*, and *Aspergillus* (Figure 2A) [164,165]. ITS profiling in healthy adults consistently detects *Candida* spp. and *Saccharomyces cerevisiae*, along with low-abundance environmental molds that fluctuate over time. Fungi typically account for less than 1% of gut microbial reads, yet their metabolic activity, biofilm formation, and strong engagement of PRRs mean small quantitative shifts can disproportionately affect mucosal and systemic immunity [166].

Stool sequencing in adults with asthma has shown increased relative abundance of *Candida, Malassezia*, *Saccharomyces*, and *Aspergillus* compared with non-asthmatic controls, along with altered community structure and, in some cohorts, higher fungal richness. Overrepresentation of *Candida* spp., especially *C. albicans*, is reproducibly associated with exacerbation-prone phenotypes and markers of systemic inflammation, whereas lower *Candida* dominance characterizes milder disease or remission [154,167]. Heterogeneous sequencing targets, analytic pipelines, and controls for diet, medication, and geography prevent defining a single asthma mycobiome signature [168]. The early-life window is the most informative, since it coincides with immune maturation and the establishment of stable microbial communities [169]: infants who later develop asthma or wheeze show high *Candida* abundance, low fungal diversity, and unstable community composition in the first months of life, often with depletion of butyrate-producing Firmicutes, and these patterns are shaped by delivery mode, breastfeeding, antibiotic exposure, and environmental fungal burden [155,170,171]. Children who remain asthma-free have more diverse and temporally stable mycobiomes with greater *Saccharomyces* representation. In adults, severe or uncontrolled disease is associated with increased *Candida* and *Malassezia*, reduced mycobiome stability, and tighter co-occurrence networks between fungi and pathobiont bacteria. Whether these configurations drive persistent airway inflammation or follow from disease and its treatment with corticosteroids, antibiotics, and proton-pump inhibitors remains unresolved, given scarce longitudinal interventional data [172].

Intestinal fungi can influence pulmonary allergic inflammation in experimental models: intestinal overgrowth of *Wallemia mellicola* exacerbates murine asthma through Dectin-2-dependent recognition [173]. In antibiotic-treated mice, intestinal *Candida* overgrowth increased circulating PGE2, promoting alternative activation of alveolar macrophages and enhancing allergic airway inflammation [174]. However, PGE2 has cell- and context-dependent effects: in human ILC2s, it suppresses proliferation and IL-5/IL-13 production through EP2 and EP4 receptors rather than uniformly amplifying type 2 immunity [175]. Separately, elevated neonatal fecal 12,13-diHOME was associated with subsequent childhood atopy or asthma, while mechanistic experiments showed production by bacterial epoxide hydrolases and impaired immune tolerance; these findings do not establish a fungal source for this metabolite [176]. These observations suggest potential links between gut microbial changes, pulmonary inflammatory patterns, and clinical phenotypes (Figure 2D), but do not establish a single fungal metabolic pathway defining human asthma endotypes.

Integrated microbial and host profiling may help distinguish transient fungal detection from biologically relevant airway changes. Longitudinal pediatric data associate upper-airway fungal profiles with subsequent loss of asthma control, but clinical prediction requires external validation [160]. Future studies should prioritize standardized sampling, low-biomass contamination controls, and prospective links between fungal measurements and treatment outcomes rather than infer clinical utility from cross-sectional associations.

## 5. Fungi as an Emerging Threat in Asthma—From Exposure to Molecular Endotypes

Fungal exposure encompasses a broader range of taxa than those included in routine allergy testing. Clinical assessment commonly focuses on *Alternaria*, *Aspergillus*, *Cladosporium*, and *Penicillium*, whereas environmental studies reveal substantially greater fungal diversity [85,177,178]. The clinical relevance of many less-studied taxa remains uncertain. Exposure characteristics and the quality of diagnostic reagents are therefore important when interpreting fungal sensitization in asthma [9,10,85].

### 5.1. Fungal Exposure and Exposure–Response Relationships

Fungal spores are the numerically dominant class of immunologically active bioaerosol. Volumetric surveys place outdoor spore concentrations two to three orders of magnitude above pollen counts, with concentrations routinely 10^3^–10^6^ spores/m^3^ during peak sporulation in essentially every habitable region [177,179,180,181]. A systematic review of 74 European long-term observational studies reported median peak concentrations of 18,827 spores/m^3^ for *Cladosporium* and 665 spores/m^3^ for *Alternaria*, exceeding the symptomatic thresholds of 3000 and 100 spores/m^3^ on most days of the spore season [182]. Parallel aircraft and ground sampling detect spores in the free troposphere, indicating that long-range atmospheric transport sustains background exposure far from primary emission sources [183]. Indoor air forms a quantitatively distinct compartment. Dampness arising from leaks, condensation, flooding, or inadequate ventilation is the principal determinant of indoor fungal growth and associated respiratory risk. Visible mold, mold odor, and water damage are independently associated with respiratory symptoms, allergic sensitization, and asthma [134,135,184,185]. The dominant indoor genera, *Aspergillus*, *Penicillium*, *Cladosporium*, *Alternaria*, and *Fusarium*, overlap with the major outdoor allergenic taxa, and their relative abundances are modulated by building age, ventilation regime, occupant density, and local microclimate (Figure 3A) [134,186,187].

Cohorts that quantified exposure volumetrically or molecularly, rather than relying on reported dampness, have refined the exposure–response relationship. In the Cincinnati Childhood Allergy and Air Pollution Study, a high Environmental Relative Moldiness Index at age 1 predicted asthma at age 7, most strongly among atopic children [187]. Culturable airborne *Penicillium* concentrations were associated with wheezing (OR 2.12) and greater asthma severity (OR 1.99) in *Penicillium*-sensitized children, and indoor total culturable fungi were associated with emergency department visits in mold-sensitized asthmatics [188,189]. Among 644 pediatric asthmatics in the Melbourne Air Pollen Children and Adolescent study, total outdoor spore counts and concentrations of *Alternaria*, *Leptosphaeria*, *Coprinus*, and *Drechslera* were independently associated with asthma hospitalization, and *Alternaria* sensitization remained among the strongest predictors of epidemic thunderstorm asthma [190,191]. A meta-analysis of 21 case–control studies and 11 cohort studies associated childhood exposure to mouldy homes with asthma, with pooled odds ratios of 1.53 (95% CI 1.42–1.65) and 1.15 (95% CI 1.01–1.31), respectively [192]. Indoor dampness and mold account for roughly 21% of current asthma cases in the developed-country housing stock [185].

Dose-dependent associations between fungal load and asthma morbidity persist after adjusting for IgE status because cell-wall polysaccharides, proteases, and secondary metabolites act on the epithelium via pattern-recognition receptors, PAR-2 cleavage, and Th17 polarization without requiring classical sensitization [56,91,134,187]. Submicronic fungal fragments, which, under certain humidity regimes, outnumber intact spores by one to two orders of magnitude, may therefore have biological relevance comparable to whole spores [193,194]. Clinical translation is constrained by unharmonized sampling methods and the absence of validated concentration thresholds for genera other than *Alternaria* and *Cladosporium* [179,182,195]. Amplicon sequencing of the indoor mycobiome recovers taxa absent from routine monitoring, including basidiomycetes, rusts (*Pucciniales*), and powdery mildews (*Erysiphales*), whose allergenic potential is documented but unquantified at the population level [196,197,198]. Atopy, sensitization profile, host genotype (*CARD9, TLR* variants), and microbiome status further shape these relationships [53,56].

**Figure 3:**
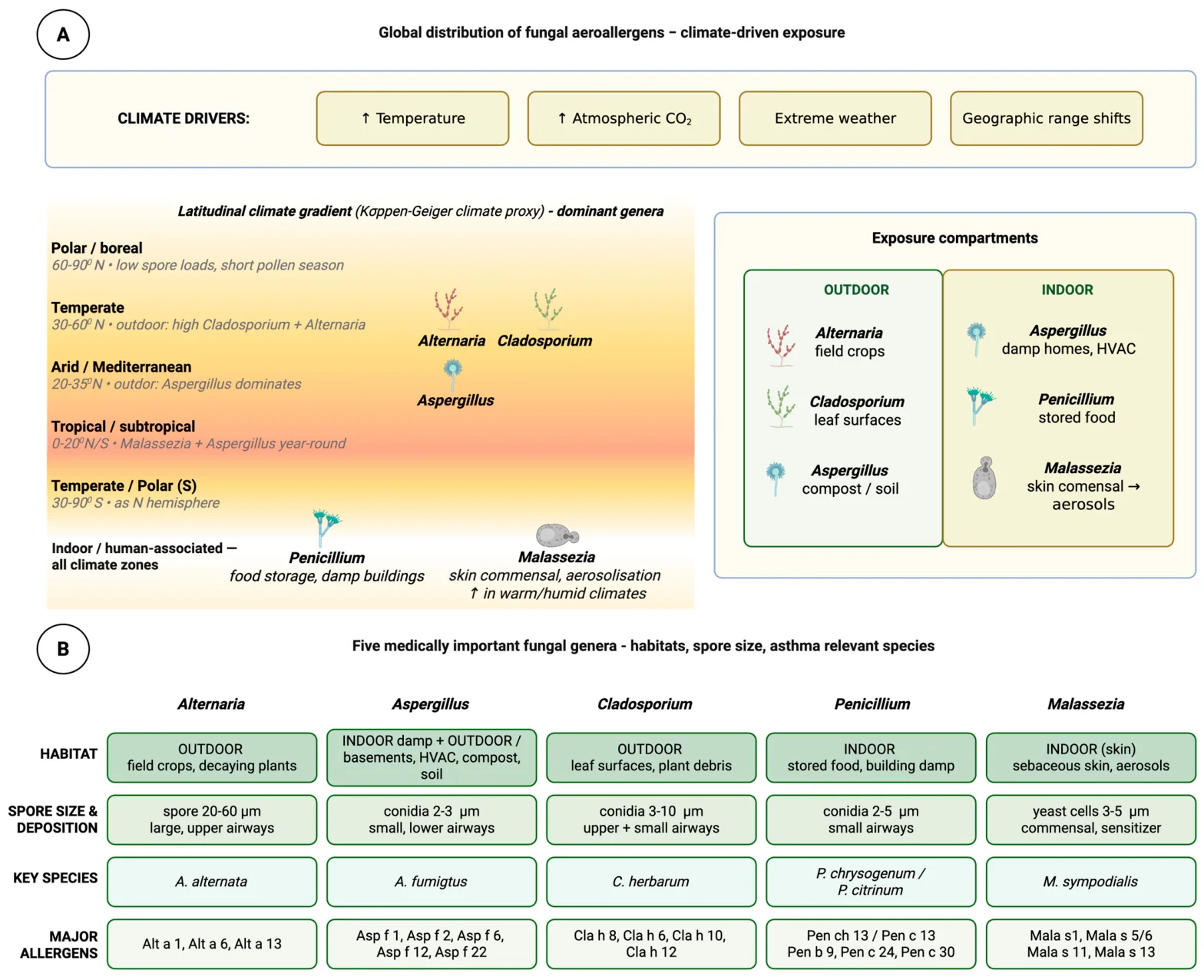
Global distribution and major genera of fungal aeroallergens. (**A**) A Köppen–Geiger climate proxy shows regions where spores of *Alternaria*, *Cladosporium*, *Aspergillus*, and *Penicillium*, and cells of *Malassezia,* are commonly detected in ambient bioaerosols, along with four climate-related drivers of exposure: rising temperature, increasing atmospheric CO_2_, extreme weather events, and geographic range shifts [199,200,201,202]. (**B**) Principal habitats, spore characteristics, and asthma-relevant species of five medically important genera, with size-dependent airway deposition. Large *Alternaria* spores (20–60 µm) deposit mainly in the nose and upper airways; *Cladosporium*, *Aspergillus*, and *Penicillium* conidia and *Malassezia* yeast cells (2–10 µm) reach the conducting airways; submicronic fragments (<1 µm) penetrate the small airways and alveoli and can constitute a substantial allergen burden when intact spore counts are low [182,184,188,189,190,191,192,194,203]. (Created in BioRender. Szuster-Ciesielska, A. (2026) https://BioRender.com/xh5hg71, accessed on 22 August 2026).

### 5.2. Major Allergenic Genera and Their Key Allergens

The immunological burden of fungal asthma is disproportionately attributable to a narrow taxonomic core. Sensitization surveys, environmental sampling campaigns, and curated allergen repositories converge on five genera, *Alternaria*, *Aspergillus*, *Cladosporium*, *Penicillium*, and *Malassezia*, as the dominant sources of IgE-reactive proteins in allergic asthma and SAFS (Figure 3B) [85,177,178,204]. The WHO/IUIS Allergen Nomenclature Sub-Committee lists more than one hundred officially recognized fungal allergens across these and a few additional genera (*Candida*, *Fusarium*, *Trichophyton*, *Curvularia*). This molecular characterization has moved fungal allergology from an extract-based discipline to a structurally informed one [178,204]. Diagnostic panels in routine use remain confined to a small subset of these molecules, and the ecological prominence of basidiomycetes and of pathogenic ascomycetes such as the powdery mildews (Erysiphales) is poorly reflected in available reagents (Figure 4A) [85,177].

*Alternaria alternata* is the most clinically important outdoor allergenic fungus in temperate climates and the genus most consistently associated with asthma severity, exacerbations, and mortality [85,204]. Its major allergen, Alt a 1, a 30 kDa β-barrel dimer with no known mammalian homolog, is recognized by 80–98% of *Alternaria*-allergic patients and serves as a species-specific marker in component-resolved diagnostics (CRD). The panel also includes Alt a 6 (enolase), Alt a 8 (mannitol dehydrogenase), Alt a 10 (aldehyde dehydrogenase), Alt a 13 (glutathione-S-transferase), the heat-shock protein Alt a 3, and the disulfide isomerase Alt a 4 [178,204]. *Alternaria* extracts also contain serine and aspartic proteases that disrupt epithelial tight junctions, activate protease-activated receptor 2, and elicit the release of TSLP, IL-25, and IL-33 independently of IgE. Thus, the dose–response associations recorded for airborne *Alternaria* reflect IgE-mediated responses to Alt a 1 acting alongside protease-driven epithelial activation [91,193].

The spectrum of *Aspergillus*-related disease, particularly *A. fumigatus*, ranges from simple IgE sensitization through SAFS to ABPA. Asp f 1, an 18 kDa cytotoxic ribonuclease, and Asp f 2, a 37 kDa cell-wall protein, are highly specific markers of ABPA, whereas Asp f 3 (peroxisomal protein), Asp f 4, and Asp f 6 (Mn-superoxide dismutase) are associated with disease severity and with ABPA complicating cystic fibrosis [9,178,204]. Several *Aspergillus* components behave as panallergens: Asp f 6 cross-reacts with human Mn-superoxide dismutase and has been implicated in atopic dermatitis, while Asp f 8 (ribosomal P2 protein) and Asp f 22 (enolase) share epitopes with homologs in *Cladosporium*, *Alternaria*, and *Penicillium*, as well as with the latex enolase Hev b 9, providing a structural basis for the cross-genus IgE reactivity seen in fungus-sensitized asthmatics [205,206]. Studies using human IgE monoclonal antibodies have shown appreciable inter-batch variability in the abundance of key allergenic proteins in commercial *Aspergillus* extracts, raising questions about the reliability of extract-based skin-prick testing [207].

*Cladosporium herbarum* and *C. cladosporioides*, among the most abundant airborne fungi worldwide, contribute to perennial and seasonal sensitization through Cla h 8 (mannitol dehydrogenase, recognized by approximately 57% of *Cladosporium*-allergic patients), Cla h 5 (acid ribosomal protein P2) and Cla h 9 (vacuolar serine protease), Cla h 6 (enolase), and Cla h 12 (acid ribosomal protein P1) [9,204]. The dominant indoor *Penicillium* species, *P. chrysogenum* and *P. citrinum,* produce Pen ch 13 and Pen c 13, alkaline serine proteases with strong IgE-binding activity and direct epithelial-activating capacity via PAR-2 cleavage [128,149,204]. *Malassezia*, traditionally linked to atopic dermatitis, has been increasingly implicated in airway sensitization in adult asthma, with Mala s 1, Mala s 5, Mala s 6 (cyclophilin), Mala s 11 (Mn-superoxide dismutase), and Mala s 13 (thioredoxin) as the principal IgE-reactive components [55,204]. Cross-reactivity among phylogenetically distant fungi is driven predominantly by conserved scaffolds, including enolases (Asp f 22, Alt a 6, Cla h 6, Hev b 9), heat-shock proteins, thioredoxins, Mn-superoxide dismutase, and cyclophilins, and only marginally by species-specific epitopes [205,206].

CRD can therefore distinguish genuine species-specific sensitization to Alt a 1 or to Asp f 1 and Asp f 2 from panallergen-driven cross-reactivity, thereby refining endotypic classification and guiding allergen-specific immunotherapy [9,178,207]. Available panels remain skewed toward *Alternaria* and *Aspergillus*, while clinically validated components for *Malassezia*, *Fusarium*, *Candida*, and the majority of basidiomycetes, rusts (Pucciniales), and powdery mildews are incomplete or absent, leaving a substantial fraction of fungus-sensitized patients without an identifiable causative allergen [177,179,208,209]. The batch-to-batch variability demonstrated for *Aspergillus* extracts likely extends to genera still tested with extracts and contributes to the inter-laboratory discordance observed in fungal IgE testing [207,210]. The relative contributions of IgE-binding proteins, non-protein fungal components such as β-glucans, chitin, mycotoxins, and extracellular vesicles, and of intact spores versus submicronic fragments remain insufficiently mapped onto current diagnostic frameworks [91,134,187,193,194].

**Figure 4:**
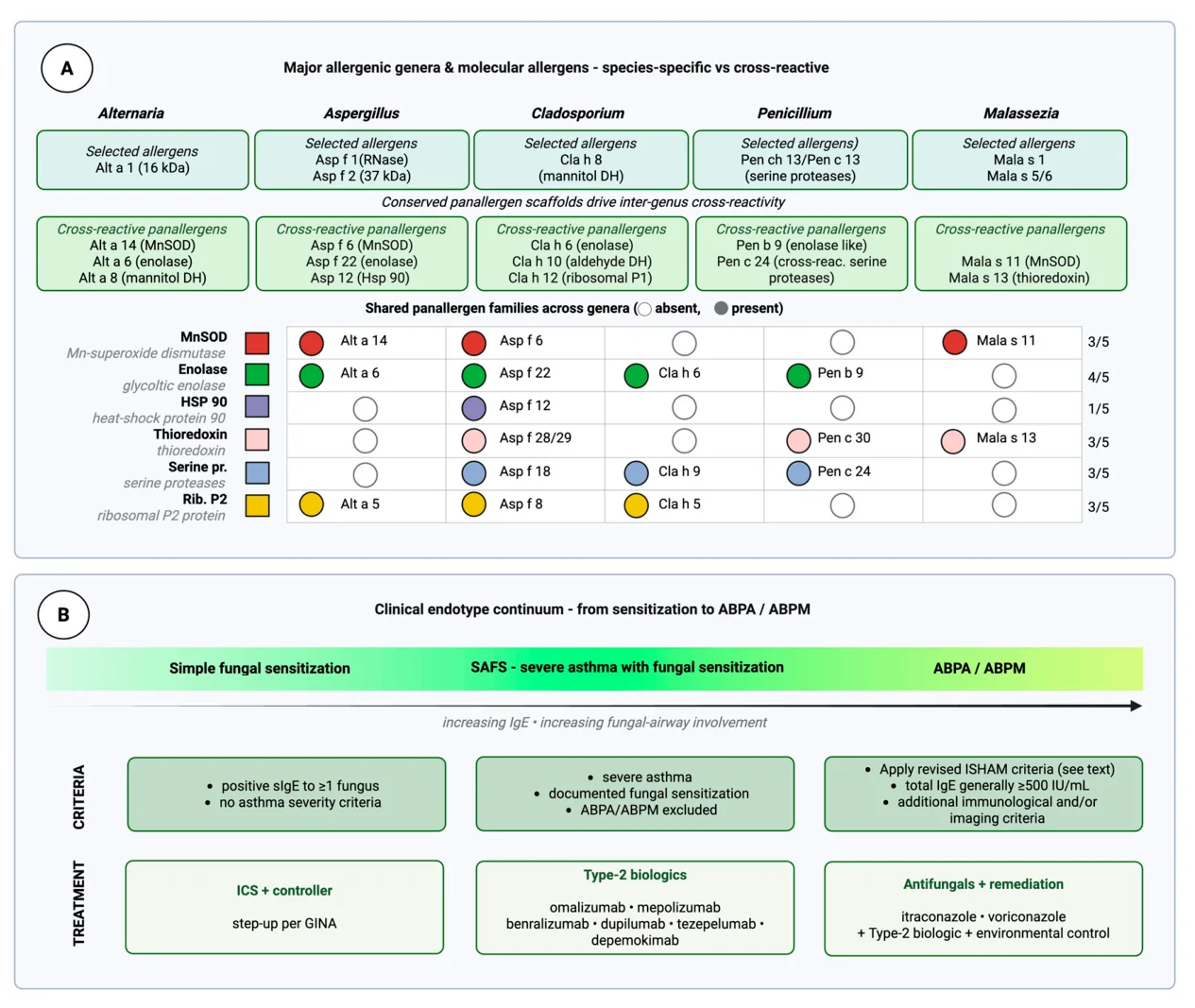
Molecular allergens and clinical endotypes of fungal asthma. (**A**) Selected fungal allergens (upper boxes) and examples of conserved protein families (lower matrix) illustrate molecular targets for sensitization studies and potential sources of IgE cross-reactivity. The presence/absence matrix summarizes six scaffolds: glycolytic enolase (documented in 4/5 genera), Mn-superoxide dismutase, thioredoxin, cross-reactive serine proteases and ribosomal P2 protein (3/5 each), and Hsp 90, currently represented mainly by *Aspergillus* Asp f 12 (1/5). Persistent gaps include extract-batch variability, underrepresentation of *Malassezia*, *Cladosporium*, and *Penicillium* on commercial panels, and the paucity of validated reagents for phytopathogenic microfungi [9,178,204,205,206,207,210]. (**B**) The clinical endotype continuum extends from simple fungal sensitization through SAFS to ABPA and ABPM, formalized by the revised ISHAM-ABPA criteria and increasingly grouped under the umbrella concept of allergic fungal airway disease (AFAD) [9,85,211,212,213]. Management comprises inhaled corticosteroids, type 2-targeted biologics (omalizumab, mepolizumab, benralizumab, dupilumab, tezepelumab, depemokimab), antifungal agents (itraconazole, voriconazole), and environmental remediation [9,213,214,215,216]. (Created in BioRender. Szuster-Ciesielska, A. (2026) https://BioRender.com/5o0dw70, accessed on 22 August 2026).

### 5.3. Severe Asthma with Fungal Sensitization and Related Entities: SAFS and ABPA

A spectrum of severe, difficult-to-treat asthma phenotypes is unified by demonstrable fungal sensitization or colonization [9,85,90,211,217]. At one end lies SAFS, introduced by Denning and colleagues for patients with severe, poorly controlled asthma and IgE-mediated sensitization to one or more fungi, but without the radiological and serological hallmarks of ABPA [9,85]. At the other end lies ABPA, characterized by airway colonization with *Aspergillus fumigatus*, intense IgE- and IgG-mediated responses, central bronchiectasis, mucus plugging, and eosinophilia [85,211,217]. The EAACI Task Force on fungal-driven airway dis-immunity, together with reappraisals that position ABPA as a treatable trait within the global asthma burden, has recast these entities as overlapping nodes along a continuum of allergic fungal airway disease (AFAD). In this continuum, SAFS, ABPA, allergic fungal rhinosinusitis, and chronic eosinophilic pneumonia share immunopathological substrates while differing in the depth of mucosal involvement, the contribution of fungal viability, and the dominant cytokine signature (Figure 4B) [211,213,217,218].

SAFS describes severe asthma with demonstrable fungal sensitization after exclusion of ABPA or ABPM; total IgE alone is insufficient to distinguish these conditions [9,85,212]. The revised ISHAM criteria allow ABPA diagnosis in patients with a predisposing condition or a compatible clinical–radiological presentation. Essential components are *A. fumigatus*-specific IgE ≥ 0.35 kUA/L and total IgE generally ≥500 IU/mL, together with at least two of the following: elevated *A. fumigatus*-specific IgG, blood eosinophilia, and suggestive imaging [212]. Lower total IgE may be accepted when the remaining criteria are fulfilled. Depending on the population and the breadth of the diagnostic panel, 35 to 75% of patients with severe asthma show IgE reactivity to at least one fungal allergen, and 25 to 40% meet full SAFS criteria [9,85,90]; in a contemporary Korean cohort, fungal sensitization was detected in 14.1% of patients with severe asthma, compared with 7.7% of those with non-severe disease [214]. SAFS is associated with lower FEV1, more frequent exacerbations, greater oral corticosteroid burden, and a higher prevalence of fixed airflow obstruction than in non-sensitized severe asthma, with histopathology intermediate between Th2-high eosinophilic asthma and neutrophilic, mixed-granulocytic patterns [9,53,90]. In a prospective cohort from the Bichat Hospital severe asthma program (*n* = 245), 28.2% of patients fulfilled SAFS criteria and experienced more frequent exacerbations and a less robust long-term response to type 2-targeted biologic therapy than non-sensitized patients [215].

Persistent intrabronchial colonization by *A. fumigatus* hyphae sustains antigen presentation, releases proteases and gliotoxin into the airway lumen, and drives a polyclonal IgE/IgG response with eosinophilia, mucus hypersecretion, and progressive bronchial damage [9,90,136,217]. Bronchocentric granulomatosis, mucoid impaction, central bronchiectasis, and fibrotic remodeling account for the high rates of fixed airflow obstruction in long-standing or inadequately treated disease [9,85,217]. ABPA is a staged rather than a static diagnosis, ranging from acute exacerbation through remission, exacerbation-prone, corticosteroid-dependent, and end-stage fibrotic phenotypes [217,218].

Between these poles lies a gray zone covered by the AFAD umbrella: patients with fungal-driven airway inflammation who fall short of the ISHAM criteria because total IgE is modestly below the threshold, bronchiectasis is absent, or sensitization involves non-*Aspergillus* fungi, yet who show mucus plugging, peripheral and airway eosinophilia, exacerbation-prone disease, and positive sputum or lavage cultures or molecular detection of *Aspergillus*, *Penicillium*, or other molds [211,217,218]. Allergic fungal rhinosinusitis is the upper-airway counterpart and frequently coexists with severe lower-airway disease, consistent with the united airways concept of a single inflammatory continuum across the respiratory tract [211,217,218,219]. Biomarker-defined subgroups within this continuum include patients with airway *Aspergillus* colonization despite negative classical serology, those with predominantly local IgE responses, and those with non-*Aspergillus* sensitization profiles, notably *Alternaria*-dominant severe asthma associated with thunderstorm-triggered exacerbations [9,85,90,217,218]. The AFAD framework also accommodates taxa beyond the classical big five, including basidiomycetes, rusts, and powdery mildews, whose clinical relevance has been obscured by the absence of validated diagnostic reagents.

### 5.4. Impact of Climate Change on Fungal Spores and Allergy Season

Airborne fungal biomass is no longer a quasi-stationary exposure variable. Anthropogenic warming, altered precipitation regimes, rising atmospheric CO_2_, more frequent extreme weather, and the geographic redistribution of host plants are reshaping fungal aerobiology on a continental scale (Figure 3A) [199,200,201,202,217,220,221]. In a longitudinal analysis of airborne spore data from 55 US National Allergy Bureau stations between 2003 and 2022, the public-health-relevant spore season began on average 22 days earlier than two decades before, with the ecologically defined onset advancing by about 11 days, and each shift correlated with rising temperatures and altered precipitation [199]. This advancement matters for *Alternaria*- and *Cladosporium*-driven asthma, whose peaks fall in late summer and autumn, the periods most affected by the extension of the growing season [162,199,217,221]. Total annual spore loads have not risen uniformly, and droughts have reduced peak concentrations in parts of the continental US, leading to earlier but not necessarily greater exposure that conventional risk models based on integrated spore counts do not capture [199]. Geographic redistribution accompanies these temporal shifts. Metabarcoding data combined with maximum entropy modeling project that the climatically suitable habitat of *A. fumigatus* could expand by approximately 77.5% under the SSP585 high-emissions scenario by 2100, exposing an additional 9 million people in Europe to environments compatible with its persistence, while *Aspergillus flavus* gains roughly 16% more territory, moving north into northern China, Russia, Scandinavia, and Alaska as parts of Africa and South America become inhospitable [200]. The northward migration reaches European latitudes, including Poland, where fungal sensitization is already common [10,217]. Redistribution of plant-pathogenic and saprophytic fungi follows that of their host plants and exposes previously unexposed populations to novel sensitizing taxa, as documented for autumnal birch rust as a newly recognized trigger of seasonal airway symptoms in northern Europe [198], which motivates our characterization of phytopathogenic microfungi as inhalant allergens [208,209,222,223].

Climate also affects spore biology. Elevated atmospheric CO_2_ enhances sporulation in several allergenic genera, increasing per-plant and per-unit-area spore output without geographic expansion [201]. Spores produced at higher temperatures are smaller and penetrate the small airways more efficiently [193,194,201]. Thunderstorms concentrate, fragment, and osmotically rupture spores and pollen grains into respirable particles implicated in epidemic thunderstorm asthma, and the frequency and geographic range of such events are projected to increase [202]. Ambient particulate matter and NO_2_ co-deposit with spores, increasing their immunogenicity [201,220,224]. Post-hurricane and post-flood housing surveys document elevated indoor spore concentrations and a shift toward more allergenic species [201].

To address these challenges, we propose integrating longer-season airborne fungal monitoring with molecular identification, meteorological records, and asthma exacerbation data. Monitoring strategies should be tailored to regional exposure patterns and, where relevant, complemented by assessments of indoor dampness. Analyses should account for both short-term peaks and cumulative exposure. This approach could inform regionally tailored exposure forecasts and guide the selection of fungal taxa for clinical validation.

## 6. Our Studies—Common Plant Fungal Parasites with High Allergenic Potential

This section synthesizes our published studies on seven plant-associated fungal and oomycete taxa collected in eastern Poland [208,209,222,223,225,226,227]; it presents no previously unpublished experimental data. The studies combined biochemical characterization with respiratory epithelial assays and murine models of allergic airway disease. Their purpose was to investigate taxa poorly represented in routine allergy assessment, not to establish clinical diagnoses or treatment indications. Figure 5 provides a qualitative, hypothesis-generating synthesis, and Supplementary Table S1 lists measured and unavailable endpoints. Clinical relevance requires confirmation in exposed human populations.

### 6.1. Identification and Characterization of New Microfungi Species Isolated from the Polish Environment

Phytopathogenic microfungi colonizing cultivated and ornamental plants are an underexplored yet biologically plausible source of inhaled allergens. Rust fungi (*Pucciniales*), powdery mildews (*Erysiphaceae*), and oomycete fungus-like organisms (*Peronosporales*, *Albuginales*) produce enormous quantities of airborne spores during well-defined seasonal windows, late spring through autumn in temperate Central Europe. Urediniospores of *Pucciniales* typically measure 12–30 μm; conidia of *Erysiphaceae* 25–45 × 10–20 μm; and sporangia of Peronosporales 15–30 μm [181,228]. These sizes place them within the aerodynamic range for efficient deposition in the upper and lower respiratory tract, with smaller fractions and spore fragments reaching the bronchiolar and alveolar regions [180,193,194].

This distinction between intact spores and their fragments is mechanistically significant. Intact urediniospores and *Erysiphaceae* conidia (12–45 μm) are efficiently intercepted in the nasal cavity and upper conducting airways, and only their smaller size fractions penetrate beyond the tracheobronchial region. *Peronosporales* sporangia in the 15–30 μm range sit at the upper limit of thoracic penetration and, in intact form, deposit predominantly in the extrathoracic and large-airway compartments. Controlled aerosolization studies of fungal material have shown that mechanical disturbance of colonized surfaces, hyphal desiccation, and spore rupture generate submicronic fragments (<1 μm) that outnumber intact spores by up to two to three orders of magnitude in the airborne fraction and carry allergenic and β-glucan-associated activity comparable to that of whole spores per unit protein [193,194]. Because these submicronic fragments follow the aerodynamic behavior of fine and ultrafine particulate matter, they bypass upper-airway filtration and deposit efficiently in the bronchiolar and alveolar compartments, where they can engage alveolar macrophages, dendritic cells, and epithelial PRRs that are anatomically inaccessible to intact 15–45 μm particles. This shift in effective size distribution plausibly explains why phytopathogenic taxa with nominally supra-respirable spore sizes, including the seven species characterized here, can still elicit distal-airway sensitization and inflammation.

None of these taxa are routinely included in clinical sensitization panels, and their immunological profiles have, until recently, been largely undescribed, a knowledge gap repeatedly highlighted in reviews of fungal allergy [177,178,204]. Historical work on basidiomycete extracts demonstrated significant skin-test reactivity and even bronchospasm in response to basidiospores [196,197], and very recent clinical observations have confirmed that exposure to a single rust species, *Melampsoridium betulinum* (birch rust), can elicit IgE responses in over three-quarters of tested patients during autumnal sporulation peaks [198]. We selected seven species recurrent in the Lublin region of eastern Poland, infecting widely cultivated host plants (plum, raspberry, cucurbits, lesser celandine, bindweed, amaranth, and ornamental hosts of *Erysiphe palczewskii*), and built a uniform pipeline for taxonomic confirmation, spore harvesting, protein extraction under non-denaturing conditions, and downstream immunological and biochemical interrogation. The isolates were collected from a single agroecological setting in eastern Poland. Whether their biochemical and immunological properties are representative of isolates from other regions or host plants requires independent validation. Our characterization of these species was guided by the recognition that a candidate respiratory allergen should ideally satisfy four converging lines of evidence: (i) demonstrable IgE-binding components or homologs of established fungal allergens, (ii) the capacity to activate respiratory epithelium at non-cytotoxic doses representative of the upper bound of likely environmental exposure, (iii) induction of allergen-specific IgE in a controlled sensitization model, and (iv) reproduction of the cellular, cytokine, and histological signature of allergic airway disease in vivo.

For *E. palczewskii* and *E. convolvuli*, we coupled this pipeline with in-depth biochemical profiling, including LC/MS-based proteomics, fatty-acid composition, and carbohydrate analysis [225]. Proteomic interrogation identified 1118 peptides in *E. palczewskii* (68.8% unique to the species) and 770 in *E. convolvuli* (47% unique). Prominent among these were ribosomal proteins, heat shock proteins (Hsp70, Hsp90), enolase, fumarate reductase, nucleoside diphosphate kinase, glycerol dehydrogenase, malate dehydrogenase, and a Rho GDP dissociation inhibitor. Several of these, particularly Hsp70/Hsp90 family members (homologous to Asp f 12) and the glycolytic enzyme enolase (homologous to Cla h 6 and Alt a 6), have well-documented allergenic counterparts across phylogenetically distant fungal genera, with experimentally confirmed cross-reactive IgE epitopes among fungal, yeast, and even latex enolases [205,206], providing a structural basis for both de novo IgE recognition and clinically meaningful cross-reactivity with already-characterized panallergens. The fatty-acid profile of both species was dominated by 18:2 linoleic acid (~30% of total fatty acids in *E. palczewskii*), the parent ω-6 polyunsaturated fatty acid from which arachidonic acid and its downstream eicosanoid mediators are generated in mammalian cells. Whether fungal-derived linoleic acid in our extracts contributes meaningfully to host eicosanoid biosynthesis, or whether the spores themselves carry preformed oxylipins with pro-inflammatory activity, remains an open question, since eicosanoid output was not directly interrogated in the functional assays and warrants dedicated follow-up. The carbohydrate analysis was dominated by hexoses, pentoses, and hexosamines, consistent with chitin- and β-glucan-derived fragments which are C-type lectin receptor ligands. These data identify a biochemical inventory compatible with innate sensing, adaptive priming, and eicosanoid-mediated inflammation.

### 6.2. In Vitro and In Vivo Evidence of Allergenic and Pro-Inflammatory Potential

Functional validation of the new candidate allergens began with two parallel in vitro studies using human bronchial (BEAS-2B) and alveolar (A549) epithelial cell lines, chosen as reductionist models of the upper and lower respiratory epithelium. In the first study, extracts of the rust fungi *Tranzschelia pruni-spinosae* and *Phragmidium rubi-idaei*, applied at sub-cytotoxic concentrations, significantly increased IL-1β and GM-CSF production in both cell lines, produced a measurable rise in intracellular reactive oxygen species, and elevated matrix metalloproteinase secretion, a pattern that, although modest for any single parameter, converged on disruption of tight junction integrity, with loss of E-cadherin and occludin signals on immunofluorescence [208]. In the second, methodologically matched study, extracts of the oomycete fungus-like organisms *Peronospora ficariae* and *Wilsoniana bliti* produced a more dissociated profile: *W. bliti* triggered only marginal responses, whereas *P. ficariae* significantly induced IL-1β and GM-CSF in both BEAS-2B and A549 cells, accompanied by ROS generation, MMPs release, and degradation of E-cadherin and occludin [209]. The selective activity of *P. ficariae* over *W. bliti*, despite their close ecological niche, illustrates that allergenic potential cannot be inferred from taxonomic proximity alone and reinforces the need for species-level functional screening. This dissociation supports a per-species rather than per-genus or per-order approach to allergen panel design and parallels the well-documented intra-genus heterogeneity observed for *Aspergillus* species, where *A. fumigatus* dominates clinical allergy despite being only one of many *Aspergillus* species encountered in the environment [10,207].

The convergence on IL-1β, GM-CSF, ROS, MMPs, and barrier disruption is mechanistically informative. It mirrors the four-step model of epithelial alarmin release, innate amplification, and tight junction breakdown that we summarized for *Alternaria* and *Aspergillus* proteases, and provides in vitro evidence that previously overlooked phytopathogenic taxa engage the same alarmin–protease–barrier axis. Epithelial barrier dysfunction is now recognized as an upstream amplifier of both type-2 and type-17 inflammation and as a putative driver of corticosteroid-resistant endotypes. Although we did not formally test CLR dependence using receptor blockade or knockout systems, the cytokine signature (IL-1β, GM-CSF) observed across all species and both cell lines is consistent with Dectin-1/Syk–CARD9 engagement by the β-glucan- and chitin-rich cell wall fragments identified in our biochemical analysis.

To test whether these in vitro signals translate into a coherent allergic airway disease in vivo, we used a BALB/c mouse model of acute and chronic asthma. Animals were sensitized by intraperitoneal injection of a fungal extract emulsified in aluminum hydroxide and then challenged intranasally. Ovalbumin served as a positive reference allergen, and PBS/adjuvant as negative controls. Readouts included differential blood leukocyte counts, serum cytokine profiling (IL-4, IL-5, IL-13, TNF-α, TGF-β by ELISA), allergen-specific IgE titers, and histopathology of formalin-fixed lungs.

In the first in vivo study, *T. pruni-spinosae* and *P. rubi-idaei* induced significant increases in all five measured cytokines, with magnitudes that, in several cases, matched or exceeded those of ovalbumin: in the acute model, both rusts produced marked increases in serum IL-5 and IL-13, along with a marked rise in TGF-β, characteristic of the remodeling-associated subepithelial fibrosis; in the chronic model, *T. pruni-spinosae* generated the highest IL-5 concentration of any group tested. Both fungi elicited robust allergen-specific IgE titers, and serum IgE correlated strongly with IL-4 levels, consistent with classical Th2-driven class switching. Histology showed peribronchial eosinophil infiltration, goblet cell hyperplasia, and progressive airway wall thickening in chronically exposed animals [223].

A second in vivo study, using the same protocol and extracts from the powdery mildew *Podosphaera fusca* and the downy mildew *Peronospora ficariae*, replicated and extended these findings. Both species induced significant production of IL-4, IL-5, IL-13, TNF-α, and TGF-β; elevated allergen-specific IgE; and the characteristic histopathological signature of acute and chronic experimental asthma, including eosinophilic infiltrates and goblet cell hyperplasia. The magnitude of the response was modestly lower than that obtained with ovalbumin, but the consistency across cytokine, serological, and histological endpoints argues that *P. fusca* and *P. ficariae* should be regarded as *bona fide*, if comparatively moderate, inducers of allergic airway disease [222]. We then extended the same experimental framework to two powdery mildews of the genus *Erysiphe*. Intranasal challenge with *E. palczewskii* and *E. convolvuli* extracts in sensitized mice produced highly significant elevations in IL-4, IL-5, IL-13, TNF-α, and TGF-β in both acute and chronic models, robust allergen-specific IgE responses, and histopathological evidence of eosinophilic airway inflammation, goblet cell hyperplasia, and airway wall remodeling, thereby completing the in vivo phenotype anticipated by the biochemical analysis of these species [227].

Together, these published studies provide preclinical evidence of allergenic and pro-inflammatory potential among the plant-associated fungus taxa examined. The extent of characterization varies by species, and the findings do not establish their clinical allergenicity in humans.

### 6.3. Integration of Epidemiologic Data with Molecular Findings—Positioning New Fungi Within Existing Endotypes of Fungal Asthma

*Pucciniales* and *Erysiphaceae* are among the most prolific spore producers in temperate agricultural and peri-urban landscapes. Their airborne concentrations overlap and, during peak sporulation, can exceed those of canonical aeroallergens such as *Cladosporium* and *Alternaria*. Their seasonal peaks—late spring to early summer for *Erysiphe* and *Podosphaera*, midsummer to autumn for *Tranzschelia* and *Phragmidium*, and early spring for *Peronospora ficariae*—coincide with well-documented exacerbations in patients with allergic asthma and chronic rhinosinusitis, including those who remain seronegative on standard fungal panels. Whether sensitization to these taxa contributes to seasonal exacerbations in patients who test negative on standard fungal panels remains to be determined.

When we map our data onto the endotype framework used in this review, three patterns stand out. First, the rust fungi *T. pruni-spinosae* and *P. rubi-idaei* elicit the most pronounced IL-5 and IgE responses among all species tested, accompanied by histological evidence of eosinophilic infiltration and goblet cell hyperplasia. The cytokine and histological findings are consistent with type 2 allergic airway inflammation in mice. However, these experimental models do not establish equivalence to human SAFS or predict responses to targeted therapies. Their relevance to specific clinical phenotypes requires validation in human studies [9]. The translational relevance of our findings will therefore depend on dedicated clinical studies that test sensitization to *Pucciniales*-derived allergens in well-characterized SAFS cohorts. Second, increased TGF-β and subepithelial thickening in the murine experiments support a remodeling-associated response, but do not establish a contribution to fixed airflow obstruction in exposed patients. Third, the epithelial responses observed in vitro support investigating barrier dysfunction as a potential mechanism; their contribution to human asthma endotypes remains to be established.

Figure 5 provides a qualitative, hypothesis-generating synthesis of these findings. The horizontal and vertical axes represent conceptual spectra of inflammatory polarization and structural pathobiology, respectively. The approximate placement of each taxon is based on convergence across directly measured epithelial, cytokine, serological, and histopathological findings reported in the primary studies [208,209,222,223,225,226,227]. This is not a composite score or quantitative ranking. Supplementary Table S1 summarizes the available observations and clearly identifies outcomes that were not determined. Missing values were not imputed.

These observations support prioritizing rust and powdery mildew proteins, including candidate heat-shock proteins, enolase, and other metabolic enzymes, for human sensitization and cross-reactivity studies before considering their inclusion in routine diagnostic panels. Given the conserved nature of Hsp70/Hsp90 and enolase epitopes, sensitization to *Pucciniales* and *Erysiphaceae* may also generate false-positive signals in *Aspergillus*- and *Alternaria*-based component assays. Conversely, a subset of patients currently classified as Alt a 6- or Cla h 6-sensitized may, in fact, be primarily sensitized through environmental exposure to aeroallergens from rust and powdery mildew, a hypothesis amenable to direct testing in IgE cross-inhibition assays [205,206].

Epidemiologically, future airway mycobiome studies, particularly the ITS-based and shotgun metagenomic surveys, should explicitly resolve sequences from *Pucciniales*, *Erysiphaceae*, and *Peronosporales* rather than collapsing them into higher-rank “unclassified Basidiomycota/Ascomycota/Oomycota” bins, so that exposure to these taxa can be quantitatively linked to endotype assignment, exacerbation frequency, and response to biologic therapy. Mechanistically, the convergence of independent species on the alarmin–barrier–Th2–remodeling axis suggests that we should redraw the boundaries of the currently recognized fungal asthma endotypes around mechanism rather than a fixed, historically biased species list. Our work on these seven taxa is intended as a first step in that direction and as a methodological template, including uniform biochemical characterization, paired in vitro epithelial assays, and a standardized murine sensitization–challenge protocol, that can be applied systematically to the long tail of environmental microfungi whose contribution to human respiratory allergy remains largely uncharted.

### 6.4. Limitations and Translational Caveats

Several methodological caveats should be kept in mind when interpreting the body of work summarized above. First, all functional assays used whole soluble protein extracts rather than intact, environmentally dispersed spores; the contributions of spore-surface components, particulate effects, and germination-associated factors to the in vivo allergenic potential of these species therefore remain to be defined. Second, BALB/c mice are constitutively Th2-skewed and provide a sensitive but biased readout of allergen potency, which may overestimate the magnitude of type-2 responses observed in less polarized hosts. Third, we achieved sensitization by intraperitoneal injection in aluminum hydroxide, a route that bypasses mucosal priming that occurs naturally on the airway and gut epithelium and may not fully recapitulate the kinetics of human sensitization. Fourth, we did not quantify eicosanoid mediators (PGD2, LTC4, cysLTs) in either epithelial supernatants or bronchoalveolar lavage (BAL) fluid, leaving the fatty-acid–driven component of inflammation hypothetical at this stage. Fifth, our isolates come from a single agroecological setting in eastern Poland; we have not yet addressed potential intra-species variability across geographic regions and host plants. Finally, we have not tested human samples to date; confirming clinical relevance through skin-prick testing and component-resolved IgE assays in patients with seasonal allergic asthma is the necessary translational next step. Converging evidence across biochemical, in vitro, and in vivo levels in seven independent species nonetheless provides a mechanistic foundation for the prioritized translational follow-up outlined above.

## 7. Molecularly Targeted Approaches to Asthma Treatment and Management in the Context of Fungal Sensitization

Therapy for fungal-sensitized asthma must interrupt, at defined molecular nodes, the type-2 and mixed type-2/type-17 cascades initiated by fungal exposure, rather than control bronchoconstriction alone. Pharmacotherapy has shifted from near-universal reliance on inhaled corticosteroids and bronchodilators toward biologic agents that neutralize individual cytokines, cytokine receptors, or upstream epithelial alarmins, and, in defined subgroups, toward combined antifungal chemotherapy, allergen immunotherapy, and environmental remediation. Corticosteroid-induced immunosuppression, persistent antigenic exposure, and mixed inflammatory endotypes of fungal disease pose challenges that paradigms developed for allergen-monomorphic Th2-high asthma do not address. Table 1 presents a concise framework for the diagnostic and therapeutic evaluation of fungal-sensitized severe asthma.

This framework is intended as a practical synthesis of the evidence reviewed in this article, not as a substitute for current severe-asthma or ABPA/ABPM guidelines. Biologic eligibility, dosing, and sequencing should follow applicable regulatory criteria and local recommendations. 

### 7.1. Current Standard Pharmacotherapy

Asthma management centers on Inhaled corticosteroids (ICS)-containing regimens, supplemented as needed with long-acting bronchodilators and other controller therapies, and may include maintenance-and-reliever treatment with ICS–formoterol. Short-acting β2-agonist monotherapy is no longer recommended because it leaves airway inflammation untreated and increases the risk of severe exacerbations [229]. ICS suppress airway inflammation through glucocorticoid receptor-mediated regulation of pro- and anti-inflammatory gene expression [253] and improve control and lung function while reducing exacerbations and the need for systemic corticosteroids [254,255]. However, responses are less consistent in severe, neutrophilic, or mixed type-2/type-17 disease, and local and systemic adverse effects accumulate with prolonged or high-dose exposure [256]. Corticosteroid treatment has also been associated with an increased filamentous fungal burden in the airways [250], so repeated escalation in inadequately controlled patients may sustain fungal persistence. Fixed-dose ICS/LABA combinations, particularly formoterol-based maintenance-and-reliever regimens, reduce exacerbations and reliever use more effectively than increasing the ICS dose alone [257,258,259]. Repeated or prolonged oral corticosteroid exposure carries substantial cumulative morbidity and healthcare burden [230,260], making steroid-sparing strategies relevant in SAFS and ABPA. None of these agents eliminates fungal exposure, colonization, or the antigen-specific immune response. Supplementary Table S2 summarizes the pharmacological characteristics, benefits, limitations, and adverse effects of each class.

### 7.2. Biologic Therapies Targeting Type-2 Inflammation

Most patients with severe asthma show a type-2 inflammatory pattern driven by IgE, IL-4, IL-5, IL-13, and the upstream epithelial alarmins TSLP, IL-33, and IL-25, which has directed development toward agents that neutralize individual nodes of this network [261,262]; an eosinophilic phenotype is estimated in more than 80% of real-world severe-asthma cohorts [262]. Fungal sensitization, defined by skin-prick or specific IgE reactivity, is documented in up to 80% of patients with severe asthma and is more prevalent in corticosteroid-dependent disease [91,217]. This overlap supports extending type-2-directed biologics to the fungal-sensitized subgroup and explains the incomplete responses observed, in which persistent fungal antigenic drive and mixed type-2/type-17 inflammation compete with blockade of single downstream cytokines. Table 2 summarizes the approved agents and the evidence available for fungal-sensitized disease.

Omalizumab, the first biologic approved for asthma, is a humanized IgG1κ antibody that binds free IgE, prevents its interaction with FcεRI, downregulates the receptor on basophils and mast cells by up to 99% and 90%, and interrupts mediator release [263]. When added to conventional therapy, it reduces exacerbations and permits ICS step-down. Adverse events are dominated by injection-site reactions, headache, and upper respiratory symptoms, with no excess of serious events across 14 systematic reviews and meta-analyses, although rare anaphylaxis has been documented [264]. In ABPA complicating asthma or cystic fibrosis, open-label series and small randomized studies report reduced exacerbations, lower systemic corticosteroid requirements, and lower IgE-guided disease activity, but the very high baseline IgE concentrations typical of ABPA exceed the range from which standard dosing tables were derived [240,241,265]. In SAFS without overt ABPA, evidence from post hoc analyses of pivotal trials and real-world cohorts favors patients with unequivocal IgE-mediated sensitization to a defined fungal allergen over those with polysensitization or a predominantly non-IgE-mediated fungal drive.

IL-5 governs eosinophil growth, maturation, survival, and recruitment, and its blockade rapidly and durably depletes blood and airway eosinophils. Mepolizumab and reslizumab neutralize free IL-5 and impair the JAK–STAT, Btk, and Ras/Raf-ERK signaling required for eosinophil activation, reducing oral corticosteroid burden and exacerbation frequency while improving quality of life. Safety is supported by a meta-analysis of ten randomized trials in 4471 patients and by real-world effectiveness studies. Transient creatine kinase elevation and myalgia are characteristic of reslizumab, and severe anaphylaxis is rare in both [266,267,268,269,270]. Benralizumab targets the IL-5Rα subunit, preventing IL-5 Rα/IL-5Rβ heterodimerization and, through FcγRIIIa engagement on neutrophils, macrophages, and natural killer cells, producing antibody-dependent cytotoxic depletion of eosinophils. Long-term European and Asian real-world data confirm sustained control, oral corticosteroid tapering, better quality of life, and fewer asthma- related hospitalizations, and use has been extended to children aged 6–11 years [271,272,273,274,275]. Depemokimab carries a triple Fc substitution that increases affinity for the neonatal Fc receptor and extends dosing to six- month intervals. Approval followed pooled SWIFT-1 and SWIFT-2 data showing a 72% reduction in exacerbations requiring hospitalization, with injection-site reactions predominating and a class-level warning for severe reactions [276,277,278]. A Phase III program in children aged 6–11 years is scheduled for the second half of 2026 (NCT 07671001, https://clinicaltrials.gov/study/NCT07671001, accessed on 15 August 2026). In SAFS, mepolizumab and benralizumab reduce exacerbation rates and oral corticosteroid use in predominantly eosinophilic patients, with response magnitude comparable to the broader severe- eosinophilic population [239,242]; in ABPA, open- label series show reductions in exacerbations, corticosteroid burden, and total IgE, but randomized data remain scarce, and expert consensus restricts use to eosinophilic ABPA phenotypes with inadequate response to azoles and corticosteroids [242,243].

Dupilumab, a fully human IgG4κ antibody against IL-4Rα, is the only agent in practice that blocks signaling by IL-4 and IL-13, the cytokines that drive IgE class-switching, mucus hypersecretion, and remodeling in the type-2-high airway [244]. Injection-site reactions and transient eosinophilia are the most frequent adverse events, a small increase in parasitic infections has been seen in children, and real-world data confirm sustained control with fewer annual exacerbations [279,280,281]. Evidence in ABPA includes case-series observations [245]. Separately, post hoc analyses of QUEST and its TRAVERSE extension support dupilumab efficacy in patients with uncontrolled moderate-to-severe asthma and fungal sensitization [246]. Rademikibart, an IL-4Rα-directed IgG4κ antibody with higher preclinical binding affinity than dupilumab [282,283], is under Phase 2 and Phase 3 evaluation as add-on therapy in type-2 asthma and in acute exacerbations of type-2-driven asthma or COPD (NCT06940141 (https://clinicaltrials.gov/study/NCT06940141?viewType=Card&cond=Asthma&aggFilters=status:act%20rec%20not&page=25&rank=247, accessed on 15 August 2026), NCT07705737 (https://clinicaltrials.gov/study/NCT07705737?viewType=, accessed on 15 August 2026), NCT06488755 (https://clinicaltrials.gov/study/NCT06488755?viewType=, accessed on 15 August 2026).

Attention has shifted from downstream effector cytokines to epithelial alarmins that initiate and amplify type-2 inflammation, which is relevant to fungal disease because fungal proteases and structural components trigger alarmin release from bronchial epithelial cells [131,284]. Tezepelumab, a fully human IgG2λ antibody, binds TSLP and prevents formation of the TSLPR–IL-7Rα complex, thereby blocking activation of eosinophils, mast cells, dendritic cells, ILC2s, and Th2 cells and reducing IL-4, IL-5, IL-9, and IL-13 output [285]; acting above the type-2/non-type-2 dichotomy, it retains efficacy in non-eosinophilic and low-T2 phenotypes, with arthralgia and pharyngitis as the most frequent adverse events [247,286,287]. Randomized and subgroup analyses support tezepelumab efficacy in severe, uncontrolled asthma [248,249]. These analyses do not constitute dedicated real-world evidence in SAFS or establish a preferred biologic for this population.

Several anti-alarmin agents are in late-phase development. The anti-IL-33 antibody tozorakimab has been evaluated in Phase 2 COMETA (NCT06897748, https://clinicaltrials.gov/study/NCT06897748, accessed on 15 August 2026) and FRONTIER-3 (NCT04570657, https://clinicaltrials.gov/study/NCT04570657, accessed on 15 August 2026) and is advancing in Phase 3 ROMEO (NCT07566195, https://clinicaltrials.gov/study/NCT07566195, accessed on 15 August 2026) and Phase 2b UMBRIEL (NCT06932263, https://clinicaltrials.gov/study/NCT06932263, accessed on 15 August 2026), both expected to complete in 2028. Itepekimab, a second anti-IL-33 antibody, reduced COPD exacerbations in AERIFY-1 (NCT04701983, https://clinicaltrials.gov/study/NCT04701983, accessed on 15 August 2026) but not in AERIFY-2 (NCT04751487, https://clinicaltrials.gov/study/NCT04751487, accessed on 15 August 2026), a discrepancy likely to delay authorization and slow asthma development. GB-0895, an anti-TSLP antibody with a half-life enabling six-monthly subcutaneous dosing, entered Phase 3 in late 2025 and early 2026 (SOLAIRIA-1 and SOLAIRIA-2, NCT07276724 (https://clinicaltrials.gov/study/NCT07276724, accessed on 15 August 2026), NCT07359846 (https://clinicaltrials.gov/study/NCT07359846, accessed on 15 August 2026) after a machine-learning-driven design program that compressed development to four years, with readouts anticipated in late 2028 to early 2029. Tilrekimig (PF-07275315), a trispecific antibody against TSLP, IL-4, and IL-13, is in Phase 2 asthma trials (NCT06977581, https://cdek.pharmacy.purdue.edu/trial/NCT06977581/, accessed on 15 August 2026) and a Phase 2/3 COPD program (NCT07363694, https://clinicaltrials.gov/study/NCT07363694, accessed on 15 August 2026). Verekitug, an antibody targeting TSLPR, more potent than tezepelumab in preclinical work [288], allows 12- to 24-week dosing and entered Phase 2 in 2025 (NCT06966479, https://clinicaltrials.gov/study/NCT06966479, accessed on 15 August 2026). These agents act where the airway epithelium translates fungal exposure into type-2 amplification and may therefore modify disease activity even when the effector-cytokine cascade is mixed. However, confirmatory trials in fungal-sensitized cohorts are lacking, and the evidence base remains subgroup analyses of registrational studies in general severe-asthma populations.

**Table 2:** Approved biologic therapies for severe asthma: mechanism, dosing, and evidence in fungal-sensitized disease. SC—subcutaneous, IV—intravenous

Biologic	Target	Approval	Dosing	Mechanism (Summary)	Evidence in Fungal-Sensitized Asthma/ABPA	Key References
Omalizumab	Free IgE	FDA 2003; EMA 2005	SC 75–600 mg every 2 or 4 weeks (IgE- and weight-adjusted)	Prevents IgE binding to FcεRI on mast cells and basophils; downregulates FcεRI expression.	Reduces exacerbations and OCS burden in ABPA (open-label series, RCTs limited); role in SAFS supported for defined single-allergen sensitization.	[240,263,265]
Mepolizumab	IL-5	FDA & EMA 2015	SC 100 mg every 4 weeks (adults/adolescents); 40 mg (6–11 y)	Binds free IL-5, preventing engagement of IL-5Rα and downstream JAK–STAT, Btk, Ras/Raf-ERK signaling.	Open-label series in ABPA report reduced exacerbations, OCS, and total IgE; effective in eosinophilic SAFS.	[242,243,266,267,289]
Reslizumab	IL-5	FDA & EMA 2016	IV 3 mg/kg every 4 weeks (adults only)	Binds free IL-5, blocking IL-5Rα engagement.	Limited fungal-specific data; effective in eosinophilic severe asthma overall.	[268,269]
Benralizumab	IL-5Rα	FDA/EMA 2017–2018; extended to 6–11 y (US 2024)	SC 30 mg (≥35 kg) or 10 mg (<35 kg) every 4 weeks × 3, then every 8 weeks	Prevents IL-5Rα/Rβ heterodimerization; Fc region engages FcγRIIIa → ADCC-mediated eosinophil apoptosis.	Reduces exacerbations and OCS use in eosinophilic SAFS; case reports in ABPA.	[239,271,272,273,274,275]
Depemokimab	IL-5	FDA Dec 2025; EMA Feb 2026	SC 100 mg every 6 months	Fc YTE modifications (positions 252, 254, 256) increase FcRn affinity and endosomal recycling, extending half-life.	No dedicated fungal-sensitized cohort published to date; program in 6–11 y planned H2 2026.	[276,277,278] NCT07671001 (https://clinicaltrials.gov/study/NCT07671001?intr=Depemokimab&viewType=Table&rank=2, accessed on 15 August 2026)
Dupilumab	IL-4Rα	FDA 2018; EMA 2019	SC 400–600 mg load, then 200–300 mg every 2 weeks (weight-based in 6–11 y)	Blocks IL-4Rα, inhibiting signaling of both IL-4 and IL-13 (IgE class-switching, mucus, remodeling).	ABPA: case-series evidence. Fungal-sensitized asthma: efficacy supported by post hoc analyses of QUEST and its TRAVERSE extension.	[244,245,246,279,281]
Tezepelumab	TSLP	FDA 2021; EMA 2022	SC 210 mg every 4 weeks	Prevents TSLP–TSLPR/IL-7Rα engagement; blocks upstream activation of ILC2s, Th2, dendritic and mast cells.	Randomized trial and subgroup evidence supports efficacy in severe, uncontrolled asthma; these cited reports do not establish a preferred role specifically in SAFS	[248,249]

### 7.3. Antifungal Therapy in Severe Asthma with Fungal Sensitization

Antifungal therapy targets fungal growth rather than the host inflammatory pathways targeted by biologics, but fungal sensitization or airway fungal isolation alone should not prompt azole treatment. When selecting treatment, clinicians should distinguish ABPA/ABPM from SAFS, as the disease-specific recommendations for ABPA/ABPM do not automatically apply to SAFS [212]. Randomized evidence in the latter setting is inconsistent: FAST reported improved asthma-related quality of life with itraconazole [13], whereas EVITA3, which randomized 65 patients to voriconazole 200 mg twice daily or placebo for 3 months followed by 9 months of observation, found no significant benefit in severe exacerbations or asthma-related quality of life [231]. These findings do not support routine antifungal treatment for patients with fungal-sensitized asthma [13,231]. When azole therapy is considered, potential adverse effects and drug interactions require careful assessment [212].

Three considerations complicate this approach. Triazoles inhibit cytochrome P450 3A4 and can increase systemic exposure to fluticasone and budesonide several-fold, leading to iatrogenic Cushing’s syndrome, adrenal suppression, and metabolic complications. Prescribing them alongside corticosteroids requires reviewing the entire regimen and, where feasible, therapeutic drug monitoring [235,236]. The international spread of azole-resistant *A. fumigatus*, driven by clinical selection and agricultural use of triazole fungicides, threatens the durability of the approach and necessitates species identification and susceptibility testing when first-line therapy fails [237,238]. The optimal duration of therapy, criteria for intensification or discontinuation, and pre-treatment predictors of response remain empirical; baseline total IgE, sputum galactomannan, radiological mucus plugging, and molecularly assessed colonization inform decisions but have not been validated as response biomarkers [212]. Inhaled delivery aims to concentrate the drug at the site of colonization while limiting systemic exposure and interactions: inhaled amphotericin B has been used empirically in ABPA, in colonization of remodeled airways, and in lung-transplant recipients, with variable tolerability [290], and inhaled itraconazole and the long-acting inhaled triazole PC945 show favorable early-phase tolerability and measurable reductions in airway *Aspergillus* burden [291,292].

### 7.4. Allergen Immunotherapy and Environmental Control Measures

Biologics and antifungals neutralize effector cytokines, deplete effector cells, or reduce the antigenic load without altering the immunological memory that sustains the allergic response. Allergen immunotherapy targets that memory by inducing regulatory T- and B-cell responses, shifting allergen-specific IgE toward IgG4-blocking antibodies, and, in successful cases, restoring tolerance [293]. For fungal allergens, immunotherapy is far less developed than for pollen or house-dust mite because fungal extracts are difficult to standardize, vary between batches, and cover few clinically relevant genera [204]. Subcutaneous immunotherapy with *A. alternata* and *C. herbarum* extracts is the most studied intervention: randomized and observational studies with *A. alternata* report reduced symptom scores, medication use, and bronchospasm in sensitized children and adults, with an acceptable safety profile in screened populations [294,295,296]. Molecular immunotherapy based on recombinant Alt a 1 has been explored preclinically and in early-phase studies to standardize exposure and limit reactions to non-allergenic extract components [297,298]. Evidence for *A. fumigatus* immunotherapy is limited and largely historical, with reports of severe systemic reactions in patients with high total IgE and airway colonization; consensus holds that classical subcutaneous *Aspergillus* immunotherapy is contraindicated in ABPA and that its role in SAFS is not established [299]. Sublingual immunotherapy for fungal allergens is under investigation but not yet supported by robust evidence in asthma [300].

Environmental control is the most universally applicable non-pharmacological measure because the intensity and composition of fungal exposure in domestic, occupational, and outdoor settings modify disease activity. Structured remediation of indoor reservoirs, including moisture control, removal of visible mold, high-efficiency particulate air filtration, and replacement of contaminated furnishings where appropriate, reduces symptom scores, exacerbation frequency, and healthcare utilization among fungal-sensitized patients [251,252]. Occupational exposure to fungal aerosols in agriculture, waste processing, and the food industry warrants hazard assessment and, where indicated, engineering controls or redeployment [301]. Outdoor exposure is less amenable to individual control but can be mitigated by avoidance guidance during peak sporulation windows, which becomes increasingly important as climate-driven shifts in spore phenology extend seasonal exposure. Surveillance and patient counseling also need to address the phytopathogenic microfungi described above, whose contribution to airborne exposure requires targeted monitoring.

### 7.5. Future Perspectives: Personalized Medicine Integrating Fungal Sensitization Profiles and Mycobiome Data

Severe fungal-sensitized asthma was until recently managed with corticosteroid dose escalation and empirical antifungal therapy, whereas biologics targeting IgE, IL-5/IL-5R, IL-4/IL-13, and TSLP are now routinely available, and additional alarmin- and receptor-directed molecules are in late-phase evaluation. Therapeutic decisions for the individual patient remain largely empirical, guided by blood eosinophil count, total and specific serum IgE, and fractional exhaled nitric oxide, biomarkers validated in cohorts in which fungal sensitization was not systematically assessed or was excluded. Personalized care in this population therefore depends on integrating fungal-specific molecular, cellular, and microbiological information into diagnostic algorithms and on prospectively validating these profiles as predictors of response.

Three developments support integration. Component-resolved diagnostics based on recombinant fungal allergens resolve the sensitization profile at the molecular level, distinguishing primary sensitization from cross-reactivity and clinically irrelevant patterns. This could stratify patients for allergen-specific interventions and identify those likely to benefit disproportionately from antifungal chemotherapy or immunotherapy against a defined molecular allergen [302,303]. Culture-independent characterization of the airway and gut mycobiome by internal transcribed spacer sequencing and shotgun metagenomics places therapeutic response in the context of fungal community composition and dynamics rather than the presence of a single culturable organism [11,304]. Preliminary studies associate specific signatures, including overrepresentation of *Malassezia*, *Aspergillus*, and *Candida*, with severe disease and with the mixed Th2/Th17 endotype that responds imperfectly to type-2-directed biologics [250,305]. Multi-omics platforms that combine transcriptomic, proteomic, and metabolomic readouts from airway samples with clinical and microbiological data are beginning to identify endotype signatures that transcend the type-2 dichotomy and capture the mixed patterns characteristic of fungal disease [306,307].

Clinical translation will require standardized diagnostic reagents for fungal taxa underrepresented in clinical mycology, including the phytopathogenic taxa characterized in our studies but for which validated skin-prick and specific IgE reagents are not commercially available. It will also require prospective trials of biologics in subgroups stratified by fungal sensitization status rather than by conventional type-2 biomarkers alone, to determine whether the attenuated long-term response suggested by observational cohorts reflects endotype-level attenuation, residual antigenic load, or a mixed Th2/Th17 pattern for which combined biologic-plus-antifungal regimens or upstream alarmin blockade would be more effective. Tezepelumab, verekitug, and other alarmin-directed agents, whose mechanism spans type-2 and non-type-2 inflammation, warrant dedicated evaluation in fungal-sensitized cohorts. Further out, the gut–lung mycobiological axis, in which changes in the intestinal fungal community modulate systemic and pulmonary immune tone, raises the possibility of targeting the mycobiome itself with narrow-spectrum antifungals, live biotherapeutic products, or dietary interventions as an upstream modulator of airway inflammation. These approaches remain conceptual, but they follow from treating fungi in asthma as active molecular and ecological agents in pathogenesis rather than accessory allergens.

## 8. Conclusions and Future Directions

Fungal sensitization is linked to severe asthma, and exposure to indoor dampness and mold is a modifiable risk factor. SAFS and ABPA must be distinguished because fungal sensitization alone does not establish the cause of persistent symptoms or determine treatment. The clinical interpretation of a positive fungal test depends on the patient’s disease phenotype and exposure history.

Experimental evidence links fungal recognition and protease activity to epithelial injury and overlapping type 2/type 17 responses. These mechanisms may help explain persistent inflammation in fungal-associated asthma. Mycobiome studies suggest that microbial interactions also influence disease expression, but most human data remain associative. Our studies of plant-associated microfungi support investigating taxa beyond those routinely considered in fungal allergy; their clinical relevance requires validation in exposed populations.

Management should combine assessment of fungal sensitization with evaluation of relevant environmental exposures in patients with difficult-to-treat asthma. Antifungal treatment has an established role in ABPA, whereas its use in SAFS requires careful patient selection. Select biologics based on clinical phenotype and established eligibility criteria. Sequencing-based tests and expanded allergen panels require validation before routine use. Reducing indoor dampness and reducing avoidable occupational fungal exposure remain important components of care.

Future studies should identify fungal markers that predict treatment benefit from a specific intervention. This requires longitudinal cohorts that distinguish transient exposure from persistent airway colonization and link these patterns to treatment response. Standardized allergen preparations are needed to assess sensitization to less-studied fungi. Biomarkers of non-IgE-mediated responses should be evaluated against clinical outcomes, including exacerbations and corticosteroid requirements. Integrated mycobiome and host-response analyses will be most useful when they address these defined clinical questions. Prospective validation should establish whether the resulting measurements add useful information to existing assessment and management.

## Figures and Tables

**Figure 1:**
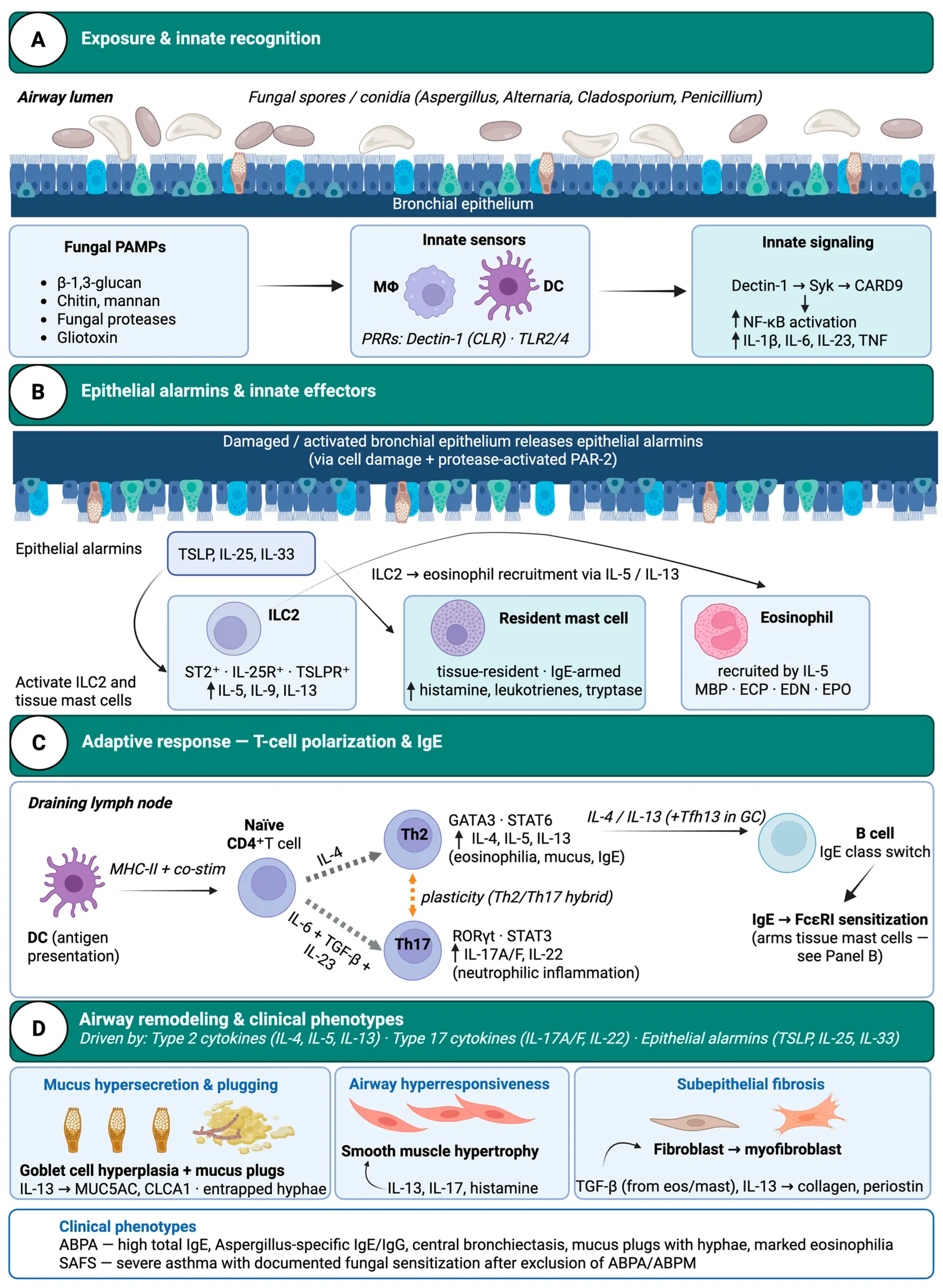
Cellular and molecular pathways in fungal asthma—from spore inhalation to airway remodeling (Created in BioRender. Szuster-Ciesielska, A. (2026) https://BioRender.com/z33upgp, accessed on 20 August 2026). (**A**) Deposited spores of *Aspergillus*, *Alternaria*, *Cladosporium*, and *Penicillium* are sensed by macrophages (Mϕ) and dendritic cells (DC) via C-type lectin and Toll-like receptors (TLRs), triggering Syk–CARD9 and NF-κB activation. (**B**) Protease-mediated PAR-2 cleavage and epithelial damage release TSLP, IL-25, and IL-33, which activate ILC2s and mast cells. (**C**) Dendritic cells presenting fungal antigen in the draining lymph node drive parallel Th2 and Th17 polarization, hybrid Th2/Th17 cells, and IgE class switching. (**D**) Sustained type-2, type-17, and alarmin signaling produce mucus plugging, smooth-muscle hypertrophy, and subepithelial fibrosis, underlying the ABPA and SAFS phenotypes.

**Figure 2:**
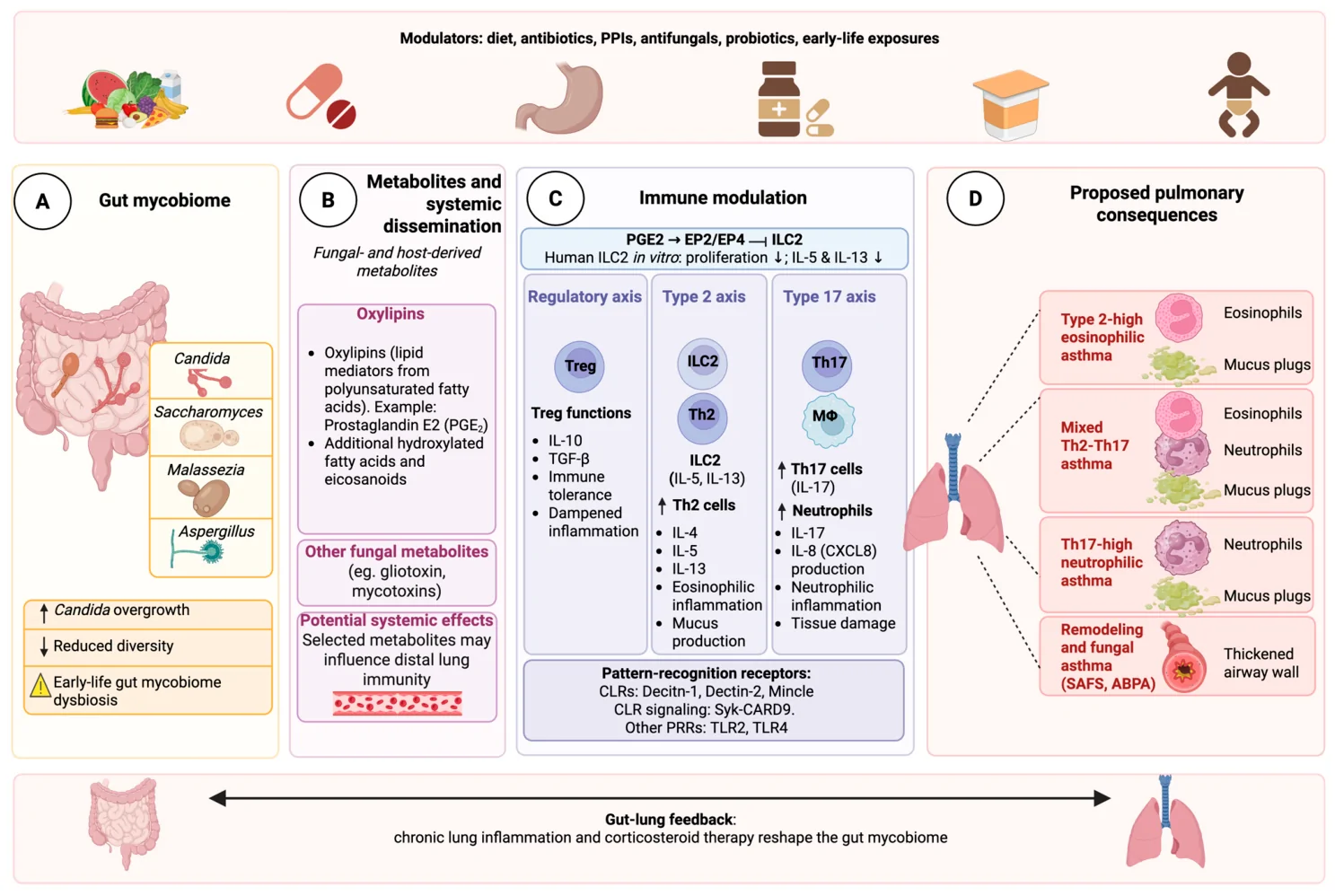
Proposed links between the gut mycobiome and airway inflammation. Created in BioRender. Szuster-Ciesielska, A. (2026) https://BioRender.com/7uj16sc, accessed on 20 August 2026. (**A**) Representative intestinal fungal taxa and examples of community alterations associated with disrupted host–microbial interactions; these changes are context-dependent rather than a universal signature of asthma. (**B**) Fungal- and host-derived metabolites, including prostaglandin E_2_ (PGE_2_), may influence distal immune responses. Their systemic availability and pulmonary effects should not be assumed to be equivalent across mediators. (**C**) Regulatory, type 2, and type 17 immune pathways potentially involved in gut–lung communication. Effects depend on the mediator, receptor, target cell, and experimental setting; notably, PGE_2_ inhibits human ILC2 proliferation and IL-5/IL-13 production via EP2/EP4 in vitro. CLR-associated Syk–CARD9 signaling is distinct from TLR2/TLR4 pathways. (**D**) Proposed pulmonary consequences, including inflammatory and remodeling-associated phenotypes. SAFS and ABPA are shown as clinical contexts, not established gut-metabolite-defined endotypes. Dashed connectors indicate proposed relationships rather than validated causal pathways in humans. The upper and lower panels summarize potential modifiers and gut–lung feedback. The schematic integrates selected observations and mechanistic hypotheses; it does not imply that all pathways occur simultaneously or are established in human asthma.

**Figure 5:**
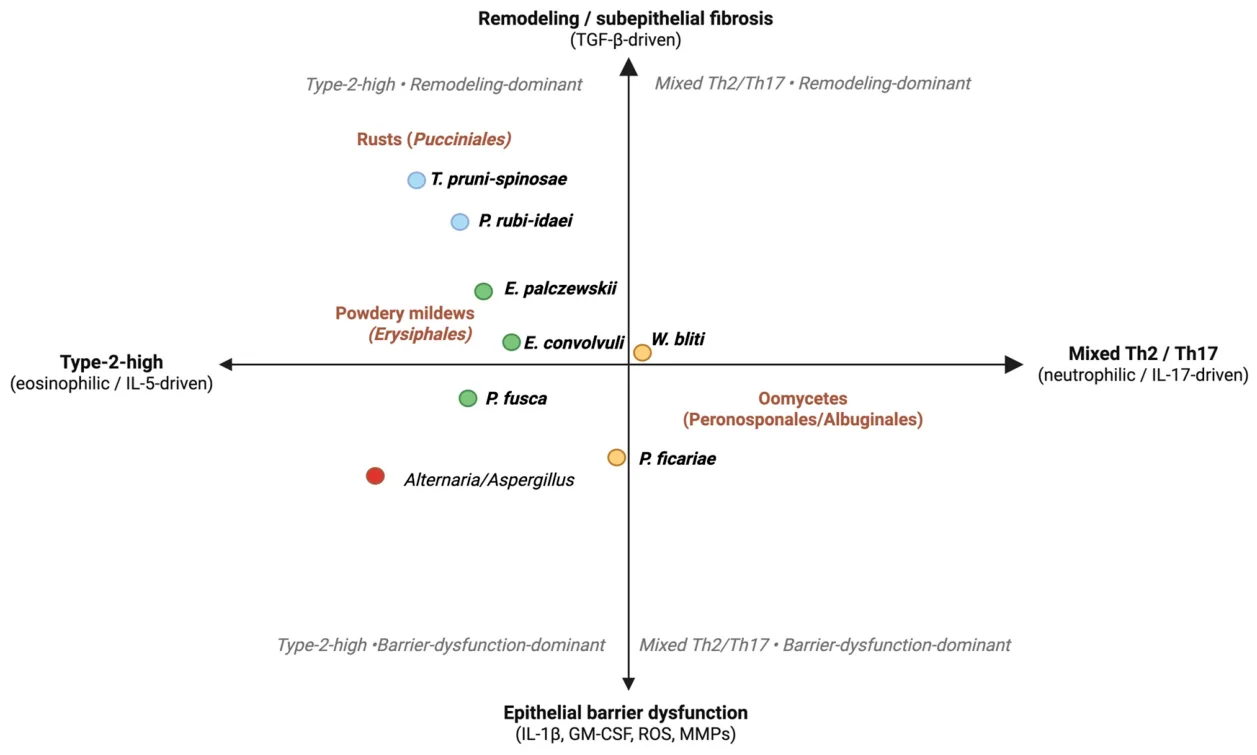
Qualitative positioning of seven phytopathogenic taxa within the fungal-asthma endotype landscape. How to read the map: movement from left to right indicates a conceptual shift from a predominantly type-2-high/eosinophilic response toward mixed Th2/Th17 inflammation, whereas movement from bottom to top indicates a shift from epithelial barrier dysfunction toward remodeling and subepithelial fibrosis. Rust fungi occupy the type-2-high/remodeling field, while powdery mildews show predominantly type-2-high profiles with variable structural effects. *P. ficariae* is positioned in the epithelial-barrier-dysfunction field, whereas *W. bliti* is near the center, reflecting limited experimental evidence rather than a defined intermediate endotype. Colors identify taxonomic groups: blue, *Pucciniales* (rust fungi); green, *Erysiphaceae* (powdery mildews); orange, *Peronosporales* and *Albuginales* (oomycete plant pathogens); and red, the canonical *Alternaria/Aspergillus* reference cluster. The approximate placement of taxa provides a qualitative, hypothesis-generating synthesis of directly measured findings and should not be interpreted as a quantitatively derived score, ranking, estimate of clinical prevalence, or measure of disease severity. Missing IL-17A/IL-13, TEER, and other experimental values were not imputed. All circles are the same size and do not encode CLR engagement or response magnitude. Supplementary Table S1 summarizes supporting observations and unavailable endpoints [208,209,222,223,225,226,227]. (Created in BioRender. Szuster-Ciesielska, A. (2026) https://BioRender.com/2a06woj, accessed on 23 August 2026).

**Table 1:** Practical diagnostic and therapeutic framework for fungal-sensitized difficult-to-treat or severe asthma.

Clinical Stage	Recommended Assessment	Interpretation or Suggested Action
Confirm difficult-to-treat or severe asthma	Confirm the diagnosis; assess adherence, inhaler technique, comorbidities, and occupational, residential, and agricultural exposures; optimize ICS-containing therapy [229,230].	Exclude modifiable causes of poor asthma control before attributing severity to fungal sensitization.
Screen for fungal sensitization	Perform skin-prick testing and/or serum-specific IgE testing for *A. fumigatus* and other locally relevant fungi. Measure total IgE and blood eosinophils [9,204,210,211,214].	A positive test indicates sensitization but does not alone establish fungal-driven disease or justify antifungal therapy [215].
Evaluate for ABPA or ABPM	In patients with *Aspergillus* sensitization, elevated total IgE, eosinophilia, mucus plugging, recurrent infiltrates, or bronchiectasis, assess *A. fumigatus*-specific IgG and obtain chest CT where clinically indicated. Apply the revised ISHAM criteria. For suspected disease caused by another fungus, consider ABPM [212,213,217].	Distinguish ABPA/ABPM from SAFS because the diagnostic criteria, strength of evidence, and treatment approach differ [212].
Identify SAFS	Confirm severe asthma and fungal sensitization after excluding ABPA/ABPM [9,13,211,214].	SAFS is a diagnosis of exclusion. Sensitization may coexist with eosinophilic, allergic, mixed-granulocytic, or remodeling-dominant disease.
Consider antifungal therapy	Antifungal treatment is established in ABPA/ABPM. In SAFS, consider an individualized triazole trial only in selected patients with persistent uncontrolled disease and evidence supporting clinically relevant fungal sensitization or airway colonization, after optimization of standard therapy [13,212,231,232,233]	Review drug interactions, hepatic function, QT-related risk, and local azole resistance; use therapeutic drug monitoring where available. Reassess clinical benefit and discontinue ineffective or poorly tolerated treatment [234,235,236,237,238].
Select biologic therapy	Match treatment to the dominant treatable trait rather than fungal sensitization alone [217,239].	Use the phenotype- and biomarker-based framework below.
Allergic/IgE-driven phenotype	Consider anti-IgE therapy when perennial allergen sensitization, IgE concentration, body weight, and the approved dosing range are appropriate.	Omalizumab has the largest published experience in allergic asthma and ABPA, although ABPA evidence is derived mainly from observational studies [240,241].
Eosinophilic phenotype	Consider anti-IL-5 or anti-IL-5R therapy in patients with elevated eosinophils, recurrent exacerbations, or corticosteroid dependence.	Particularly relevant when eosinophilia is a dominant feature of SAFS or ABPA [239,242,243].
IL-4/IL-13-dominant phenotype	Consider anti-IL-4Rα therapy in patients with elevated eosinophils and/or FeNO, corticosteroid dependence, or relevant type-2 comorbidities.	Dupilumab may be useful in mucus- and type-2-dominant disease, but evidence specific to ABPA and SAFS remains limited [244,245,246].
Broad or incompletely defined phenotype	Consider anti-TSLP therapy when exacerbations persist across biomarker ranges or when the phenotype is not adequately controlled by downstream type-2-directed therapy.	Fungal sensitization alone is insufficient to designate tezepelumab as the preferred biologic; direct comparative evidence in SAFS is lacking [216,247,248,249].
Reassessment	Monitor exacerbations, symptom control, lung function, oral corticosteroid exposure, eosinophils, total IgE where appropriate, imaging abnormalities, fungal burden, adverse effects, and environmental exposure.	Lack of meaningful response should prompt reassessment of the diagnosis, phenotype, fungal relevance, adherence, and treatment strategy [212,250,251,252].

## Data Availability

No new data were created or analyzed in this study. Data sharing is not applicable to this article.

## Supplementary Materials

The following supporting information can be downloaded at https://www.mdpi.com/article/10.3390/ijms27198639/s1.

## Abbreviations

The following abbreviations are used in this manuscript. Species and gene names, along with taxonomic authorities, are provided in the main text and are not repeated here.

**Abbreviation**	**Definition**
A549	Human alveolar epithelial cell line
ABPA	Allergic bronchopulmonary aspergillosis
ABPA-CB	ABPA with central bronchiectasis
ABPM	Allergic bronchopulmonary mycosis
AFAD	Allergic fungal airway disease
AHR	Airway hyperresponsiveness
APC	Antigen-presenting cell
ASM	Airway smooth muscle
ATLANTIS	Assessment of Small Airways Involvement in Asthma
BAL	Bronchoalveolar lavage
BALB/c	Inbred laboratory mouse strain
BEAS-2B	Human bronchial epithelial cell line
BUPAS	Belarus, Ukraine, Poland Asthma Study
CARD9	Caspase recruitment domain–containing protein 9
CASCADE	Mechanistic biopsy substudy of tezepelumab in asthma
CF	Cystic fibrosis
CHIT1	Chitotriosidase 1 gene
CI	Confidence interval
CLCA1	Calcium-activated chloride channel regulator 1
CLEC7A	C-type lectin domain containing 7A (encodes Dectin-1)
CLR	C-type lectin receptor
co-stim	Co-stimulatory signal
CO_2_	Carbon dioxide
COPD	Chronic obstructive pulmonary disease
CRD	Component-resolved diagnostics
DALY	Disability-adjusted life year
DC/DCs	Dendritic cell/dendritic cells
Dectin-1/Dectin-2	Dendritic cell–associated C-type lectin receptors 1 and 2
DESTINATION	Long-term extension study of tezepelumab
DH	Dehydrogenase
diHOME	Dihydroxyoctadecenoic acid
EAACI	European Academy of Allergy and Clinical Immunology
ECAP	Epidemiology of Allergic Diseases in Poland
ECM	Extracellular matrix
ECP	Eosinophil cationic protein
ECRHS	European Community Respiratory Health Survey
EDN	Eosinophil-derived neurotoxin
ELISA	Enzyme-linked immunosorbent assay
EMA	European Medicines Agency
EMT	Epithelial–mesenchymal transition
EPO	Eosinophil peroxidase
EVITA3	Voriconazole in Aspergillus fumigatus–associated asthma trial
FAST	Fungal Asthma Sensitization Trial (itraconazole)
FcεRI	High-affinity immunoglobulin E receptor
FDA	U.S. Food and Drug Administration
FeNO	Fractional exhaled nitric oxide
FEV_1_	Forced expiratory volume in 1 s
FLG	Filaggrin gene
FUT2	α(1,2)-fucosyltransferase 2 gene/secretor status
FVC	Forced vital capacity
GAN	Global Asthma Network
GATA3	GATA-binding protein 3
GBD	Global Burden of Disease
GBMI	Global Biobank Meta-analysis Initiative
GC	Germinal center
GINA	Global Initiative for Asthma
GM-CSF	Granulocyte–macrophage colony-stimulating factor
GWAS	Genome-wide association study
Hev b 9	Hevea brasiliensis enolase (latex allergen)
HLA-DRB1	Human leukocyte antigen DR beta 1
Hsp 90	Heat shock protein 90
HVAC	Heating, ventilation, and air conditioning
ICS	Inhaled corticosteroids
ICS/LABA	Inhaled corticosteroid/long-acting β_2_-agonist combination
IFN-γ	Interferon-gamma
IgA	Immunoglobulin A
IgE	Immunoglobulin E
IgG	Immunoglobulin G
IL	Interleukin
IL-1β	Interleukin 1 beta
IL-4	Interleukin 4
IL-4Rα	Interleukin-4 receptor alpha chain
IL-5	Interleukin 5
IL-5R	Interleukin-5 receptor
IL-6	Interleukin 6
IL-8	Interleukin 8
IL-9	Interleukin 9
IL-13	Interleukin 13
IL-17A	Interleukin 17A
IL-17F	Interleukin 17F
IL-22	Interleukin 22
IL-23	Interleukin 23
IL-25	Interleukin 25
IL-25R	Interleukin-25 receptor
IL-33	Interleukin 33
IL-36	Interleukin 36
ILC2	Group 2 innate lymphoid cell
ISAAC	International Study of Asthma and Allergies in Childhood
ISHAM	International Society for Human and Animal Mycology
ISU	ISAC standardized units
IUIS	International Union of Immunological Societies
LABA	Long-acting β_2_-agonist
LAMA	Long-acting muscarinic antagonist
LC-MS/MS	Liquid chromatography–tandem mass spectrometry
MAPCAH	Melbourne Air Pollen Children and Adolescent Health
MART	Maintenance and reliever therapy
MBP	Major basic protein
MHC-II	Major histocompatibility complex class II
Mincle	Macrophage-inducible C-type lectin
Mn-SOD	Manganese superoxide dismutase
MUC5AC	Mucin 5AC
MΦ	Macrophage
NAVIGATOR	Phase 3 trial of tezepelumab in severe, uncontrolled asthma
NCN	Polish National Science Centre (Narodowe Centrum Nauki)
NF-κB	Nuclear factor kappa-light-chain-enhancer of activated B cells
NHBE	Normal human bronchial epithelial cells
NLR	NOD-like receptor
OCS	Oral corticosteroids
ORMDL3	ORMDL sphingolipid biosynthesis regulator 3
OVA	Ovalbumin
P2	Ribosomal P2 protein
PAMP	Pathogen-associated molecular pattern
PAR-2	Protease-activated receptor 2
PGE_2_	Prostaglandin E_2_
PMSEAD	The Polish Multicentre Study of Epidemiology of Allergic Diseases
PPIs	Proton-pump inhibitors
PRR	Pattern-recognition receptor
PRS	Polygenic risk score
RNase	Ribonuclease
RORγt	Retinoic acid receptor-related orphan receptor gamma t
ROS	Reactive oxygen species
SABA	Short-acting β_2_-agonist
SAFS	Severe asthma with fungal sensitization
SAMA	Short-acting muscarinic antagonist
SDI	Socio-demographic index
SFTPA2	Surfactant protein A 2
sIgE	Allergen-specific IgE
SMC	Smooth muscle cell
SNP	Single-nucleotide polymorphism
SP-A/SP-D	Surfactant protein A/D
SPT	Skin prick test
ST2	Suppression of tumorigenicity 2 (IL-33 receptor)
STAT	Signal transducer and activator of transcription
Syk	Spleen tyrosine kinase
TAGC	Trans-National Asthma Genetic Consortium
TEER	Transepithelial electrical resistance
Tfh	T follicular helper cell
Tfh13	IL-13-producing T follicular helper cell
TGF-β	Transforming growth factor beta
Th1	T-helper cell type 1
Th2	T-helper cell type 2
Th17	T-helper cell type 17
TLR	Toll-like receptor
TLR2	Toll-like receptor 2
TLR4	Toll-like receptor 4
TNF	Tumor necrosis factor
TNF-α	Tumor necrosis factor alpha
Treg	Regulatory T cell
Trx	Thioredoxin
TSLP	Thymic stromal lymphopoietin
TSLPR	Thymic stromal lymphopoietin receptor
U-BIOPRED	Unbiased Biomarkers for the Prediction of Respiratory Disease Outcomes
VEGF	Vascular endothelial growth factor
WGA-FITC	Wheat germ agglutinin conjugated to fluorescein isothiocyanate
WHO	World Health Organization
