# Supplementary material for: Fungi in Asthma: From Epidemiology and Airway Mycobiome to Molecular Mechanisms and Novel Allergenic Phytopathogens
Source: Int J Mol Sci. 2026 Sep 27;27(19):8639. doi: 10.3390/ijms27198639 (PMC13664857; doi:10.3390/ijms27198639)
Supplement: Supplementary file 1 [file ijms-27-08639-s001.zip › ijms-4497081-supplementary.pdf]

Table S1. Qualitative summary of published experimental evidence for seven phytopathogenic taxa investigated as potential inhalant allergens.

The table reports only outcomes directly measured in the cited primary studies. It does not provide normalized values, imputed results, composite scores, or quantitative rankings.

Taxon (order)	Published experimental evidence	Epithelial/innate findings	Type 2/adaptive findings	Type 17 findings	Barrier/remodeling findings	Primary reference(s)
Tranzschelia pruni-spinosae (Pucciniales)	BEAS-2B and A549 epithelial-cell models; acute and chronic BALB/c asthma models	Increased IL-1β and GM-CSF; increased ROS and MMP release	Increased IL-4, IL-5 and IL-13; allergen-specific IgE; eosinophilic infiltration and goblet-cell hyperplasia	n.d.	Loss of E-cadherin and occludin signals in vitro; increased TGF-β and progressive airway-wall thickening in vivo	[S1, S2]
Phragmidium rubi-idaei (Pucciniales)	BEAS-2B and A549 epithelial-cell models; acute and chronic BALB/c asthma models	Increased IL-1β and GM-CSF; increased ROS and MMP release	Increased IL-4, IL-5 and IL-13; allergen-specific IgE; eosinophilic infiltration and goblet-cell hyperplasia	n.d.	Loss of E-cadherin and occludin signals in vitro; increased TGF-β and progressive airway-wall thickening in vivo	[S1,S2]
Podosphaera fusca (Erysiphales)	Acute and chronic BALB/c asthma models	n.d. in respiratory epithelial-cell models	Increased IL-4, IL-5 and IL-13; allergen-specific IgE; eosinophilic infiltration and goblet-cell hyperplasia	n.d.	Increased TGF-β and histopathological changes consistent with acute and chronic experimental asthma; TEER n.d.	[S3]
Erysiphe palczewskii (Erysiphales)	Biochemical/proteomic characterization; acute and chronic BALB/c asthma models	Potential PRR ligands and allergen homologues identified biochemically; functional epithelial readouts n.d.	Increased IL-4, IL-5 and IL-13; robust allergen-specific IgE; eosinophilic inflammation and goblet-cell hyperplasia	n.d.	Increased TGF-β and histopathological evidence of airway remodeling; TEER n.d.	[S4, S5]
Erysiphe convolvuli (Erysiphales)	Biochemical/proteomic characterization; acute and chronic BALB/c asthma models	Potential PRR ligands and allergen homologues identified biochemically; functional epithelial readouts n.d.	Increased IL-4, IL-5 and IL-13; robust allergen-specific IgE; eosinophilic inflammation and goblet-cell hyperplasia	n.d.	Increased TGF-β and histopathological evidence of airway remodeling; TEER n.d.	[S4, S5]
Peronospora ficariae (Peronosporales)	BEAS-2B and A549 epithelial-cell models; acute and chronic	Increased IL-1β and GM-CSF; ROS	Increased IL-4, IL-5 and IL-13; allergen-specific IgE;	n.d.	Degradation of E-cadherin and occludin in vitro; increased	[S3, S6]

	BALB/c asthma models	generation and MMP release	eosinophilic infiltration and goblet-cell hyperplasia		TGF-β and asthma-associated histopathology in vivo	
Wilsoniana bliti (Albuginales)	BEAS-2B and A549 epithelial-cell models	Only marginal responses under the tested conditions	n.d. in vivo	n.d.	Functional barrier and remodeling endpoints n.d.; TEER n.d.	[266]

Notes

1. "n.d." indicates that the endpoint was not determined in the cited study. Missing values were not imputed.
2. Findings are summarized qualitatively because the available studies did not measure all endpoints across every taxon. Comparisons between taxa should therefore not be interpreted as a quantitative ranking.
3. IL-17A/IL-13 ratios were not systematically measured in the published in vivo studies. No taxon was therefore assigned a quantitatively defined Th17 score.
4. TEER values are not included because complete, directly comparable published measurements were unavailable across the seven taxa. Missing or ongoing measurements were not replaced with cytokine proxies.

Abbreviations

A549, human alveolar epithelial cell line; BALB/c, inbred mouse strain; BEAS-2B, human bronchial epithelial cell line; GM-CSF, granulocyte-macrophage colony-stimulating factor; IgE, immunoglobulin E; IL, interleukin; MMP, matrix metalloproteinase; n.d., not determined; PRR, pattern-recognition receptor; ROS, reactive oxygen species; TEER, transepithelial electrical resistance; TGF-β, transforming growth factor beta.

Primary refereces

[S1] Sztandera-Tymoczek et al. The Potential Proallergenic Activity of Tranzschelia pruni-spinosae and Phragmidium rubi-idaei in vitro Studies. J Inflamm Res. 2025;18:1107–1125. doi:10.2147/JIR.S497219.

[S2] Wlaz et al. Plant Microfungi Tranzschelia pruni-spinosae and Phragmidium rubi-idaei Demonstrate Allergenic Capabilities in Mouse Models of Asthma. Int J Mol Sci. 2026;27:1507. doi:10.3390/ijms27031507.

[S3] Wlaz et al. Microfungus Podosphaera fusca and the Fungus-like Organism Peronospora ficariae as Potential Inhalant Allergens in a Mouse Model of Asthma. Cells. 2025;14:914. doi:10.3390/cells14120914.

[S4] Pac-Sosinska et al. Biochemical characteristics of extracts from proallergenic microfungi Erysiphe palczewskii and Erysiphe convolvuli. BMC Genomics. 2025;26:754. doi:10.1186/s12864-025-11862-w.

[S5] Socala et al. Plant microfungi Erysiphe palczewskii and Erysiphe convolvuli exhibit allergenic potential in both acute and chronic asthma models in mice. Future Microbiol. 2026;21:813–825. doi:10.1080/17460913.2026.2686561.

[S6] Sztandera-Tymoczek et al. In vitro Study of Fungus-Like Organisms Peronospora ficariae and Wilsoniana bliti as Potential Inhalant Allergens. J Inflamm Res. 2025;18:10445–10466. doi:10.2147/JIR.S531453.

Table S2. Overview of conventional asthma pharmacotherapy.

References are numbered independently within this supplement (S1–S10) to prevent discrepancies after renumbering of the main manuscript bibliography.

Therapy	Examples	Mechanism of action	Clinical role and benefits	Limitations	Common adverse effects	References
Inhaled corticosteroids (ICS)	Beclometasone, budesonide, ciclesonide, fluticasone propionate or furoate, mometasone	Bind intracellular glucocorticoid receptors and regulate transcription of inflammatory and anti-inflammatory genes.	Foundation of asthma controller therapy. Improve symptom control and lung function and reduce exacerbations, hospitalization, and the need for systemic corticosteroids.	Response may be incomplete in severe, corticosteroid-resistant, or non-type 2 asthma. High doses should be used only when clinically indicated. In fungal-associated disease, prolonged corticosteroid exposure may increase airway fungal burden.	Oral candidiasis, dysphonia and throat irritation; prolonged high-dose exposure may cause systemic effects, including adrenal suppression, ocular effects and reduced growth velocity in children.	[S1–S5]
Long-acting β2-agonists (LABA)	Formoterol, salmeterol	Stimulate β2-adrenergic receptors on airway smooth muscle, increasing cAMP and producing prolonged bronchodilation.	Improve symptoms and lung function and reduce exacerbation risk when used with ICS.	Must not be used without ICS in asthma because LABA do not treat the underlying airway inflammation.	Tremor, palpitations, tachycardia, headache and muscle cramps; hypokalaemia is uncommon at recommended inhaled doses.	[S1,S2,S6]
ICS/LABA combinations	Budesonide–formoterol; beclometasone–formoterol; fluticasone–salmeterol; fluticasone furoate–vilanterol; mometasone–formoterol or –indacaterol	Combine anti-inflammatory corticosteroid activity with sustained β2-receptor-mediated bronchodilation.	Preferred escalation from ICS alone when asthma remains uncontrolled. ICS–formoterol may be used as maintenance-and-reliever therapy where recommended.	Only formoterol-containing combinations are suitable for maintenance-and-reliever use. Severe or corticosteroid-resistant disease may require additional therapy.	Adverse effects associated with both components, including candidiasis, dysphonia, tremor, palpitations and headache.	[S1,S2,S6–S9]
Long-acting muscarinic antagonists (LAMA)	Tiotropium; triple ICS/LABA/LAMA inhalers where approved	Block airway muscarinic receptors, predominantly M3, reducing acetylcholine-mediated bronchoconstriction.	Add-on option for patients whose asthma remains uncontrolled despite ICS/LABA; may improve lung function and modestly reduce exacerbation risk.	Do not replace ICS and provide limited direct anti-inflammatory activity.	Dry mouth; less commonly urinary retention, blurred vision or precipitation of glaucoma after ocular exposure.	[S1,S2,S6]

Leukotriene modifiers	Montelukast, zafirlukast, pranlukast; zileuton	Block CysLT1 receptors or inhibit 5-lipoxygenase, reducing leukotriene-mediated bronchoconstriction and inflammation.	Oral alternative or add-on controller; may be useful in selected patients, including those with exercise-induced bronchoconstriction or aspirin-exacerbated respiratory disease.	Generally less effective than ICS for prevention of exacerbations. Treatment response is variable.	Headache and gastrointestinal symptoms. Montelukast carries concern for neuropsychiatric effects; zileuton may cause hepatotoxicity and requires liver-function monitoring.	[S1,S2]
Systemic corticosteroids, including oral corticosteroids (OCS)	Prednisone, prednisolone, methylprednisolone, hydrocortisone, dexamethasone	Activate glucocorticoid receptors and broadly suppress systemic inflammatory gene expression.	Short courses are used for clinically significant exacerbations. Maintenance treatment should be reserved for exceptional severe disease after optimization of other therapies.	Cumulative toxicity limits repeated or long-term use; steroid-sparing strategies should be prioritized.	Mood and sleep disturbance, hyperglycaemia, hypertension and infection risk; cumulative exposure may cause weight gain, osteoporosis, adrenal suppression, cataracts and cardiovascular or metabolic complications.	[S1,S2,S10]
Short-acting β2-agonists (SABA)	Salbutamol (albuterol), terbutaline	Rapidly stimulate β2-adrenergic receptors and relax airway smooth muscle.	Provide rapid relief of bronchoconstriction. In current treatment strategies, reliever use should be accompanied by ICS-containing therapy.	SABA-only treatment is not recommended because it does not treat airway inflammation; frequent use indicates poor control and is associated with increased exacerbation risk.	Tremor, palpitations, tachycardia, headache, nervousness and hypokalaemia, particularly at high doses.	[S1,S2]
Short-acting muscarinic antagonists (SAMA)	Ipratropium bromide	Block airway muscarinic receptors and reduce acetylcholine-mediated bronchoconstriction.	Used with SABA as short-term adjunctive treatment in moderate-to-severe acute exacerbations; may improve lung function and reduce hospital admission.	Slower and less effective as a reliever than SABA; not recommended as routine maintenance monotherapy.	Dry mouth, bitter taste, cough and headache; rarely urinary retention or blurred vision after ocular exposure.	[S1,S2]

Abbreviations

AERD, aspirin-exacerbated respiratory disease; cAMP, cyclic adenosine monophosphate; CysLT1, cysteinyl leukotriene receptor 1; ICS, inhaled corticosteroid; LABA, long-acting β2-agonist; LAMA, long-acting muscarinic antagonist; OCS, oral corticosteroid; SABA, short-acting β2-agonist; SAMA, short-acting muscarinic antagonist.

Standalone references for Table S2

[S1] Global Initiative for Asthma. Global Strategy for Asthma Management and Prevention, 2026 update. Available at: https://ginasthma.org/2026-gina-strategy-report/ (accessed 5 September 2026).

[S2] Reddel HK, Bacharier LB, Bateman ED, et al. Global Initiative for Asthma Strategy 2021: executive summary and rationale for key changes. *Eur Respir J.* 2022;59:2102730. https://doi.org/10.1183/13993003.02730-2021.

[S3] Barnes PJ. How corticosteroids control inflammation: Quintiles Prize Lecture 2005. *Br J Pharmacol.* 2006;148:245–254. https://doi.org/10.1038/sj.bjp.0706736.

[S4] Axelsson I, Naumburg E, Prietsch SO, Zhang L. Inhaled corticosteroids in children with persistent asthma: effects of different drugs and delivery devices on growth. *Cochrane Database Syst Rev.* 2019;6:CD010126. https://doi.org/10.1002/14651858.CD010126.pub2.

[S5] Fraczek MG, Chishimba L, Niven RM, et al. Corticosteroid treatment is associated with increased filamentous fungal burden in allergic fungal disease. *J Allergy Clin Immunol.* 2018;142:407–414. https://doi.org/10.1016/j.jaci.2017.09.039.

[S6] Oba Y, Anwer S, Patel T, Maduke T, Dias S. Addition of long-acting beta2 agonists or long-acting muscarinic antagonists versus doubling the dose of inhaled corticosteroids in adolescents and adults with uncontrolled asthma with medium-dose ICS: a systematic review and network meta-analysis. *Cochrane Database Syst Rev.* 2023;8:CD013797. https://doi.org/10.1002/14651858.CD013797.pub2.

[S7] Zhong N, Lin J, Mehta P, et al. Real-life effectiveness of budesonide/formoterol maintenance and reliever therapy in asthma patients across Asia: SMARTASIA study. *BMC Pulm Med.* 2013;13:22. https://doi.org/10.1186/1471-2466-13-22.

[S8] Gogali A, Rovina N, Samitas K, et al. Real-Life Effectiveness of Budesonide/Formoterol as Maintenance and Reliever Treatment via the Elpenhaler Device in Patients with Asthma: The NOTOS Observational Study. *J Asthma Allergy.* 2025;18:605–618. https://doi.org/10.2147/JAA.S517932.

[S9] Stanford RH, Nagar S, Lin X, O’Connor RD. Use of ICS/LABA on asthma exacerbation risk in patients within a medical group. *J Manag Care Spec Pharm.* 2015;21:1014–1019. https://doi.org/10.18553/jmcp.2015.21.11.1014.

[S10] Voorham J, Xu X, Price DB, et al. Healthcare resource utilization and costs associated with incremental systemic corticosteroid exposure in asthma. *Allergy.* 2019;74:273–283. https://doi.org/10.1111/all.13556.
